# Validation of a Triplex Quantitative Polymerase Chain Reaction Assay for Detection and Quantification of Traditional Protein Sources, *Pisum sativum* L. and *Glycine max* (L.) Merr., in Protein Powder Mixtures

**DOI:** 10.3389/fpls.2021.661770

**Published:** 2021-05-24

**Authors:** Adam C. Faller, Dhivya Shanmughanandhan, Subramanyam Ragupathy, Yanjun Zhang, Zhengfei Lu, Peter Chang, Gary Swanson, Steven G. Newmaster

**Affiliations:** ^1^Natural Health Product Research Alliance, College of Biological Sciences, University of Guelph, Guelph, ON, Canada; ^2^Herbalife International, Torrance, CA, United States

**Keywords:** DNA, protein, quantitative polymerase chain reaction, authentication, botanicals, quality, barcoding

## Abstract

Several botanicals have been traditionally used as protein sources, including the leguminous *Pisum sativum* L. and *Glycine max* (L.) Merr. While a rich history exists of cultivating these plants for their whole, protein-rich grain, modern use as powdered supplements present a new challenge in material authentication. The absence of clear morphological identifiers of an intact plant and the existence of long, complex supply chains behoove industry to create quick, reliable analytical tools to identify the botanical source of a protein product (many of which contain multiple sources). The utility of molecular tools for plant-based protein powder authentication is gaining traction, but few validated tools exist. Multiplex quantitative polymerase chain reaction (qPCR) can provide an economical means by which sources can be identified and relative proportions quantified. We followed established guidelines for the design, optimization, and validation of qPCR assay, and developed a triplex qPCR assay that can amplify and quantify pea and soy DNA targets, normalized by a calibrator. The assay was evaluated for analytical specificity, analytical sensitivity, efficiency, precision, dynamic range, repeatability, and reproducibility. We tested the quantitative ability of the assay using pea and soy DNA mixtures, finding exceptional quantitative linearity for both targets – 0.9983 (*p* < 0.0001) for soy and 0.9915 (*p* < 0.0001) for pea. Ratios based on mass of protein powder were also tested, resulting in non-linear patterns in data that suggested the requirement of further sample preparation optimization or algorithmic correction. Variation in fragment size within different lots of commercial protein powder samples was also analyzed, revealing low SD among lots. Ultimately, this study demonstrated the utility of qPCR in the context of protein powder mixtures and highlighted key considerations to take into account for commercial implementation.

## Introduction

For generations, leguminous botanicals have played an important dietary role for humans, as a rich source of protein (Michaels, 2016). *Glycine max* (L.) Merr. (“soy” or “soybean”) and *Pisum sativum* L. (“pea”) are among the most historically important crops, instrumental in constructing the agricultural foundation of civilization, due to the ease of high-volume storage of small, dry seeds (Wessel, 1984). Soy has been a fixture in Asian cuisine for thousands of years (evidence of domestication between 7,000 and 6,600 BC in China) and has become a staple in Western countries over the past 100 years (Lee et al., 2011; Rizzo and Baroni, 2018). Pea, dating back to the Neolithic period, originated in the Mediterranean region and became a staple global food crop, serving as important dietary source of fiber, protein, and starch (Rungruangmaitree and Jiraungkoorskul, 2017; Encyclopaedia Britannica, 2019). In modern day, these plants are cultivated in much higher yield and are commercially delivered to the public in new forms, with the notable addition of dietary supplement powders. A plethora of new dietary supplements are annually introduced in the United States, with over 90,000 different products being sold in 2014 (Starr, 2015). Plant based protein powders are one of the rapidly growing markets, with valuations as high as USD 16.3 billion by 2026 with a CAGR close to 8% (Grand View Research, 2017; Persistence Market Research, 2017; Mordor Intelligence, 2018). Consumers looking for alternative sources of protein to supplement their diets, especially those who follow a restrictive diet like veganism, make up a growing population that continues to drive up demand for these products (Jallinoja et al., 2018). Soy is expected to represent the largest segment of the plant-protein market through projections to 2025 (valued at USD 6.8 billion in 2017 – expected to grow to USD 10.4 billion by 2025; Persistence Market Research, 2017). Pea protein is experiencing the fastest market growth, with value expected to pass USD 200 million by 2023 (Global Market Insights, 2018). Both these leguminous plants are being heavily investigated in an athletics context as a suitable alternative to whey protein, with the bulk of studies observing similar strength and muscular size gains in response to resistance training in conjunction with protein supplementation (Banaszek et al., 2019). Furthermore, products that contain multiple plant-based protein sources are becoming more popular, as they ensure a “complete” amino acid profile of the nine essential amino acids for human physiology that must be obtained from food (Montgomery, 2003; Hoffman and Falvo, 2004; Woolfe and Primrose, 2004; Joy et al., 2013; Kalman, 2014).

Plant-based protein powders are clearly on a steep, upward trajectory in popularity, and in rapidly growing markets where supply is racing to meet demand, there is a clear risk of economically motivated adulteration of products (Everstine et al., 2013; Johnson, 2014). In a traditional context, authentication of soy and pea crops could depend on morphological characteristics, as intact whole grains of these plants were consumed. With processed protein powders, this method is unsuitable and alternative methods are required. Unfortunately, insufficient quality control programs currently expose the industry to vulnerability, and the common techniques for protein quality assessment are outdated and indirect (Moore et al., 2010; Everstine et al., 2013; Johnson, 2014; Marinangeli et al., 2017). The Kjeldahl and combustion (Dumas) methods for measuring total protein content are based on measuring nitrogen content of a sample, are exploitable, and do not provide any identity information for ingredients (Moore et al., 2010). DNA-based techniques may help address this gap by providing information about the sources of protein in a product. Orthogonality in testing is the path to comprehensive accountability for all market fraud vulnerabilities, and ingredient identification is the missing, most critical requirement for protein powders at the moment. Furthermore, the increase in multi-source protein powder products highlights a need for testing that can, (1) identify different protein-source ingredients in a mixture, and (2) quantify the proportion of those ingredients. Different plant-based protein sources have different market values, and those market dynamics dictate the economic incentive to short-change consumers by purposefully skewing mixture proportions toward the less expensive ingredients. For example, pea protein price is higher than soy, and while difficult to generalize, overall market pricing would be 50–80% higher for pea compared to soy on the same or similar grade levels. Thus, there is an economic incentive to liberally proportion soy protein in a mixture and a reciprocal need for analytical tools that can estimate those proportions. To date, no quantitative molecular tests for protein powder mixtures have been developed.

Any authentication strategy that is to be introduced into commercial quality control programs must go through a rigorous validation process that will ensure sufficient performance, as well as reproducibility of results. While general guidelines for analytical technique validation are established by associations like AOAC, outlines for specific approaches and analytes of interest are also required (AOAC – AOAC International; Guideline Working Group, 2012). For example, some of the considerations that apply to analytical chemistry may not apply to molecular testing and vice versa. Guidelines for the validation of real-time PCR methods for molecular identification of botanicals have been proposed, some by our lab and colleagues (Newmaster et al., 2019). This involves proposed evaluation of applicability, analytical specificity, analytical sensitivity, efficiency, precision, dynamic range, repeatability, and reproducibility (Newmaster et al., 2019). For quantitative assays that are intended to be used for more than detection, other considerations may be required, and acceptable ranges of performance parameters may be tighter, as to ensure sufficient precision (Broeders et al., 2014).

An additional common recommendation in validation guideline literature for quantitative polymerase chain reaction (qPCR) assays is the evaluation of assay “practicability” (Broeders et al., 2014; Newmaster et al., 2019). When designing any assay for eventual commercial applications, consideration must be given to cost of testing, required training of staff, affordability of equipment, and complexity of operations (Newmaster et al., 2019). Minimal resource cost and maximal testing efficiency are hallmarks of a successful design (Newmaster et al., 2019). In this study, we seek to design a triplex assay for the detection and quantification of two botanical targets – soy and pea – as well as a calibrator oligonucleotide. Multiplex molecular assays clearly highlight the ideal qualities of “practical” analytical tools. Multiplex reactions hold the benefit of higher throughput of testing, less reagent consumption, and less sample consumption (Life Technologies, 2014). Three reactions take place in one well in this triplex assay, using 1/3 of the total wells that would have been used if all reactions were run in singleplex. Advantage of this 3x increase in throughput can be significant in the setting of a quality control laboratory that tests hundreds of samples a day. The more abundant the number of different target amplifications in a multiplex reaction, the greater the resulting resource savings, compared to singleplex. Additionally, inclusion of the calibrator assay in the multiplex equates to an increase in pipette precision and minimization of error (Life Technologies, 2014). Any type of pipetting errors will affect the calibrator and target assay in the same way, as the same aliquot of sample serves as template for both assays, and reaction conditions are the same.

Appropriate standards are a necessity of any analytical system that seeks to confidently correlate analyte detection with identification (Sarma et al., 2016). In molecular authentication of botanicals, this means proper botanical reference material libraries with corresponding sequence data, and appropriate physical DNA standards that represent the expected quality of DNA analytes (the latter necessary for quantification). Comprehensive reference libraries (comprised of vouchered herbaria specimens with known provenance) are essential for the design of qPCR assays (Culley, 2013; Newmaster et al., 2019). Primers and probes must be designed with intraspecific diversity in mind; single nucleotide polymorphisms (SNPs) must not prevent amplification of certain haplotypes of a target species, given the consequence of false negatives (Phillips et al., 2019). Even if a minor SNP variant still allows annealing of a primer, thermodynamic efficiency can be reduced, affecting quantitative results of an assay. Additionally, reference sequences of non-targets will provide information about variable regions where primers and probes should be designed. Superior specificity is paramount in molecular assay design, but quantitative assays also require contextual design with regards to DNA quality. This triplex assay is designed for protein powders that undergo processing, which can lead to DNA damage and degradation (Ragupathy et al., 2019). Assay design with amplicons <200 bp usually allows amplification success for expected positives of processed material, but success at different levels of degradation has to be empirically tested (Parveen et al., 2016; Faller et al., 2019). For this reason, DNA standards that reflect the expected quality and average fragment size must be used to optimize the assay and estimate relevant parameters, like efficiency and precision. DNA quality, along with other idiosyncrasies of a given matrix, must be standardized as to ensure reproducible quantitative results (Cankar et al., 2006).

Quantification can be achieved in real-time, fluorescent based assays by two methods: absolute or relative quantification. Absolute quantification involves generating a standard dilution curve using target DNA, to which unknown samples’ PCR signals can be compared. Unknown sample quantities can be interpolated from either an external standard curve (that was previously run), or an internal standard curve (run alongside test samples on the same qPCR plate; Life Technologies, 2014). Though running a new internal standard curve with each run consumes more resources, it will capture and control for more sources of technical variation that can introduce error in quantification. Relative quantification does not rely on a standard curve and measures template quantity as a fold change compared to a control. An important requirement in relative quantification is a calibrator, used to normalize fold-change estimation of the target sequence. Relative quantification is typically used to measure expression changes of a target gene, using reverse transcribed mRNA, normalized by a non-regulated reference gene. We use this method in a unique application of measuring the proportion of soy and pea targets, normalized by a synthetic calibrator. By including synthetic calibrator DNA, we can control for variation among reactions and from run-to-run (Pfaffl, 2006).

Methods of protein powder authentication that provide direct identification of ingredients serve to address a gap in current quality control programs (Thompson et al., 2020). Molecular tools that can both differentiate and quantify ingredients in mixtures will further address the need for verification of mixture products. Quantitative techniques need to be precise enough to be able to distinguish typical thresholds of 2–5% organic material, set by industry standards and botanical pharmacopeial monographs, that separates the designation between minor acceptable contaminant and adulterant (Moreton, 2015; Sarma et al., 2016).

The main goals of this study are to, (1) optimize and validate a triplex qPCR assay for the amplification of a synthetic calibrator DNA oligo, as well as target DNA that is extracted from *G max* (L.) Merr. and *P. sativum* L. botanical material; (2) evaluate the ability of the assay to make quantitative estimates of material ratios using raw, pulse-derived DNA mass mixtures, protein powder-derived DNA mass mixtures, and dry protein powder mass mixtures. A subsidiary objective is to explore the variation that exists in fragment size among different lots of raw materials, processed into protein powder *via* the same procedure. We further compare groups of lots of the same material that were processed in different years, driven by the hypothesis that years-long storage may equate to differences in DNA quality. Given an understanding of DNA degradation, we predict equal, or slightly lower average fragment size in the older material that was processed earlier. In this study, we are able to explore the influence of DNA quality on the design of a reliable, industry-applicable molecular authentication tool.

## Materials and Methods

### Sample Collection

This triplex assay was designed to amplify a DNA marker from two target species, *G. max* (L.) Merr. and *P. sativum* L., as well as an exogenous calibrator. All optimization and testing made use of a total of 33 samples of *G. max*, 21 samples of *P. sativum*, and 13 samples of non-target botanicals and organisms (*Vicia faba* L., *Zea mays* L., *Triticum aestivum* L., *Cicer arietinum* L., *Cannabis sativa* L., *Cyamopsis tetragonoloba* (L.) Taub., *Chenopodium quinoa* Willd., *Medicago sativa* L., *Salvia hispanica* L., *Cucurbita pepo* L., *Oryza sativa* L., *Bos taurus* L., and *Acheta domesticus* L.; Table 1; Supplementary Table S1). Each botanical species had a certified herbarium voucher from the OAC Herbarium located at the University of Guelph. Table 1 outlines these references and associated voucher numbers. Two matrices, fresh pulse and processed protein powder, of each target were further used for assay development. Supplementary Table S1 outlines commercial samples that were used and their associated composition and batch numbers. *Acheta domesticus* reference was collected in Ontario, Canada and identified – the University of Guelph houses the University of Guelph Insect Collection (DEBU; Supplementary Table S1). The *B. taurus* reference was obtained and verified from the Elora Research Farms Rso, Ontario, Canada (University of Guelph affiliated; Supplementary Table S1).

**Table 1:** Reference vouchers for botanical species used in assay development (target and non-target), from OAC Herbarium Collection at the University of Guelph.

Latin botanical name	Family	Sample designation	Material matrix	Herbarium voucher #
*Glycine max* (L.) Merr.	Fabaceae	Target	Leaf	43599
*Pisum sativum* L.	Fabaceae	Target	Leaf	10378
*Vicia faba* L.	Fabaceae	Non-target	Leaf	10.275
*Zea mays* L.	Poaceae	Non-target	Leaf	4143
*Triticum aestivum* L.	Poaceae	Non-target	Leaf	97062
*Cicer arietinum* L.	Fabaceae	Non-target	Leaf	10.252
*Cannabis sativa* L.	Cannabaceae	Non-target	Leaf	730687
*Cyamopsis tetragonoloba* (L.) Taub.	Fabaceae	Non-target	Leaf	1.001CT
*Chenopodium quinoa* Willd.	Amaranthaceae	Non-target	Leaf	76930
*Medicago sativa* L.	Fabaceae	Non-target	Leaf	18041
*Salvia hispanica* L.	Lamiaceae	Non-target	Leaf	99494
*Cucurbita pepo* L.	Cucurbitaceae	Non-target	Leaf	15.280
*Oryza sativa* L.	Poaceae	Non-target	Leaf	3995

### Primer and Probe Design

Design of *G. max* and *P. sativum* primer and probe sets began with *in silico* comparison of these two target reference sequences to non-target reference sequences in the NCBI GenBank database using the BLAST algorithm. The search for suitable discriminatory sequences was guided by previous studies that sought to find efficient “barcode” regions for plants (CBOL Plant Working Group, 2009; Hollingsworth et al., 2011). Sequences were compared and aligned using Clustal Omega (Madeira et al., 2019) for nuclear *ITS1-5.8S-ITS2* regions as well as the chloroplastic *rbcL*, *matK*, *trnH–psbA* spacer, *psbK–psbI* spacer, *atpF–atpH* spacer, *rpoB*, *rpoC1*, *rpoC2*, *rpoA*, and *accD*. A sequence was first evaluated for divergence from non-target species, and further screened for internal regions that both included discriminatory SNPs and areas where primers and probes could be designed to satisfy set thermodynamic parameters. The PrimerQuest tool by Integrated DNA technologies was used to design primer and probe sets based on the sequence input into the software (Integrated DNA Technologies, Coralville, IA). Several guidelines for effective primer and probe design were followed (Coyne et al., 2001; Wang and Seed, 2006). Forward and reverse primers should have similar melting temperatures (T_m_; ±2°C), be designed 18–30 bases, lack mononucleotide guanine repeats of more than four bases, and have a 35–65% GC content. The T_m_ of the probe should be 4–6°C higher than that of the primers, with similar size criteria. Annealing temperatures (T_a_) of oligos should be ≤5°C below T_m_. GC content should be 40–60% and the 5' end of the probe should not be a guanine. The size of the amplicon should be designed within a 70–150 bp range. Additionally, criteria around thermodynamic factors were taken into account such as avoidance of internal hairpin structures or homodimers (those with a ∆G value less than −9.0 Kcal/mole), as well as heterodimers between other oligos that are to be a part of the assay (Prediger, 2013). The OligoAnalyzer Tool by IDT was used to estimate possible homo and hetero secondary structure formation, *in silico* (Integrated DNA Technologies, Coralville, IA). Any unavoidable secondary structures below the −9.0 Kcal/mole ∆G threshold may be permissible if they are non-extendable heterodimers.

Once designed, PrimerQuest was used to designate fluorophores and quenchers to sequences. The soy probe was designed with the 5' 6-FAM™ fluorophore and was dual quenched with the 3' Iowa Black® FQ and internal ZEN™. The pea probe was designed with the 5' HEX™ fluorophore and was dual quenched with the 3' Iowa Black® FQ and internal ZEN™. The calibrator probe was designed with the 5' Cy5™ fluorophore and was dual quenched with the 3' Iowa Black® RQ-Sp and internal TAO™. Primers and probes were ordered in separated vials for each set and, following reconstruction with nuclease-free ddH20, were diluted into 10uM stocks. All oligos were stored at −20°C (see Table 2 for primer and probe sequences).

**Table 2:** Sequences for primers and probes of the soy, pea, and calibrator portions of the triplex assay.

Oligo	Oligo type	Sequence	Size (bp)
*G. max* ITS2 F	Primer	5'-CCG ACT TCG CCG TGA TAA A-3'	19
*G. max* ITS2 R	Primer	5'-TCG ATG GGT CCA GAA CTG A-3'	19
*G. max* ITS2	Probe	5'-/56-FAM/ATG AGC CAC /ZEN/ GCT CGA GAC CAA TC/3IABkFQ/-3'	23
*P. sativum accD* F	Primer	5'-CTC CGG ACG CAC ATA CTA TAA-3'	21
*P. sativum accD* R	Primer	5'-AGC ACT AGC TGT TAT GGA TTC T-3'	22
*P. sativum accD*	Probe	5'-/5HEX/ATG GGA TGC /ZEN/GTA GTG GGT GAG AAA /3IABkFQ/-3'	24
Calibrator F	Primer	5'-TCA GGT AGT CAT TTG TCC-3'	18
Calibrator R	Primer	5'-GAT AGG CAT ATC TCA TCT TAA C-3'	22
Calibrator	Probe	5'-/5Cy5/ACA CCA TTT /TAO/ CAT TTC TTC CAC TGT C/3IAbRQsp/-3'	25

Quencher and fluorophore information is also noted in probe sequences. F, forward primer; R, reverse primer; and P, probe.

### DNA Extraction and Quantification

All DNA extractions were carried out with the NucleoSpin® Plant II “Genomic DNA from Plant” Kit (Macherey-Nagel GmbH & Co. KG, Düren, Germany). The cetyl trimethylammonium bromide (CTAB) based PL1 lysis buffer extraction was used in all cases except in limit of detection (LOD) determination [where a sodium dodecyl sulfate (SDS) based PL2/PL3 buffer extraction was carried out in addition to CTAB to fulfill proper LOD evaluation criteria – see “Analytical sensitivity” section; Newmaster et al., 2019]. Most extractions were performed using 80 mg of dry material for each sample in the “Mini” version of the kit (60 mg for fragment size analysis experiments – see next section). Fresh pulse materials – soybeans and peas – were first ground using a pestle and mortar that was cleaned with an RNA/RNase/DNase eliminator in between uses. DNA extraction protocol was followed according to the manufacturer’s instructions except for small amendments in CTAB (1 ml of PL1 lysis buffer, 20 μl RNAse, 1 ml PC Buffer, 60 ml elution buffer) and SDS (600 μl of PL2/ 150 μl PL3 lysis buffer, 20 μl RNAse, 1 ml PC Buffer, 60 ml elution buffer) methods. Incubation during lysis for all methods was increased to 1 h with vortexing every 10 min. For mixture experiments based on dry mass ratios of powders, 1 g of material was used for extraction in the larger “Midi” Kit with a CTAB protocol (amendments: 5.25 ml PL1, 62.5 μl RNAse, 4.6 ml PC, 200 μl). DNA concentration in all eluates was fluorometrically measured using a Qubit™ 3.0 Fluorometer with a Qubit® dsDNA High Sensitivity assay kit, according to the manufacturer’s instructions (Invitrogen, Carlsbad, CA).

### Assay Optimization

Three different sets of primers and probes were optimized for amplification performance using a Roche LightCycler® 480 System (Roche Holding AG, Basel, Switzerland). All reactions used white-bottom, 96-well PCR plates and were run for 45 cycles. Optimized parameters included primer concentrations, probe concentrations, reaction volume, annealing temperature, and annealing time. The calibrator assay was previously designed by the Natural Health Product Research Alliance (NHPRA) using these criteria. To allow multiplex capability, oligo sets were optimized around the same T_a_. The assay was also designed with a shortened “two-step” amplification protocol, with annealing and elongation combined into one temperature step. Optimization began with testing forward and reverse primer concentrations at 100, 200, 500, and 800 nM in a matrix format (all combinations were run in duplicate) and selecting the curve that displayed the earliest C_t_, while retaining a high amplitude, sigmoidal shape. Probe concentrations (50, 100, 200, 250, and 300 nM) were evaluated in the same way. Next, different reaction volumes (30 and 40 μl, based on proportional increase or decrease of master mix), annealing temperature (58, 59, and 60°C) and annealing time (20, 25, and 30 s) were assessed by running standard curves (six 10x dilutions each) and determining the combinations that resulted in maximal amplification efficiency. The final triplex assay mixture included 15 μl 2X SensiFAST™ Probe No-ROX Master Mix (Bioline, London, United Kingdom), 9 μl oligos (250 nM soy probe, 500 nM each soy forward and reverse primer, 250 nM pea probe, 500 nM each pea forward and reverse primer, 100 nM calibrator probe, 200 nM each calibrator forward and reverse primer), and 6 μl space for template, for a total volume of 30 μl (Supplementary Table S2). The thermocycling protocol consisted of an initial 2 min incubation at 95°C followed by 45 cycles of 10 s denaturation at 95°C, 20 s annealing/elongation at 58°C (then 30 s cooling at 40°C). The calibrator portion of the assay was further optimized to determine the quantity of gBlock target to include as to give a reliable C_t_ among replicate reactions without interfering with amplification of pea or soy target (Integrated DNA Technologies, Coralville, IA). Higher amounts of calibrator (>0.05 ng) caused competitive amplification with the target DNA, while low concentrations were observed to be highly variable and not stable upon repeated freeze/thaw cycles. The optimal quantity of calibrator that showed consistent results with minimal influence on multiplex amplification was determined to be 0.026 ng of gBlock DNA in each reaction (Supplementary Table S2).

### Data Acquisition

Data were analyzed using the LightCycler® 480 Version 1.5 software (Roche Holding AG, Basel, Switzerland). The Second Derivative Maximum (SDM) method was used to measure the C_t_s of curves (calls C_t_s at the “maximum acceleration” point of the exponential phase of the curve). The process is automated, removing subjective determination of a threshold level (Roche Diagnostics GmbH, 2008).

Additionally, the “High Confidence” algorithm was used for C_t_ value determination. This algorithm focusses on sample curves with a notable rise and a high signal-to-noise ratio, in order to only call highly realiable C_t_s and reduce the risk of false positives. This algorithm is recommended for all experiments using color compensation (Roche Diagnostics GmbH, 2008).

A Color Compensation HexaplexPLUS LightMix® Kit (TIB MOLBIOL, Berlin, Germany) was used to remove “crosstalk” in potentially interacting fluorescent signals of adjacent FAM and HEX channels. Calibration color compensation experiments were run according to manufacturers instructions, and a “color compensation object” was created in the LightCycler® for the FAM-HEX channels. This object must be applied to each qPCR experiment file during data analysis, before C_t_s are calculated.

### Analytical Specificity

Empirical validation of the triplex assay’s analytical specificity involves screening a panel of known positive samples, as well as known negatives, and expressing results in the following percentage formats (Codex Alimentarius Commission, 2010):

%TruePositive=100×number of correctly classified known samplestotal number of known positive samples

%FalsePositive=100×number of misclassified known negative samplestotal number of known negative  samples

%TrueNegative=100×number of classified known negative samplestotal number of known negative samples

%FalseNegative=100×number of misclassified known positive samplestotal number of known positive samples

The number of false positive and false negative samples should be zero, and true positive and negative samples should both be 100%.

The soy assay was tested with 33 known positive targets consisting of different matrices (whole dry soybean, soy protein powder isolate, and soy isoflavones). The pea assay was tested with 21 known positive targets, also comprised of different matrices (whole dry pea, pea protein, pea starch, and pea fiber). Both assays were tested with 13 non-targets, representing closely related species (4/13 family level) and industry-relevant, possible adulterants (13/13; Supplementary Table S1). No-template controls were included in all runs according to validation guidelines (Bustin et al., 2009). Two microliters of each sample’s DNA extract were used in each reaction.

### Assay Performance

Assay performance includes evaluation of PCR efficiency, analytical sensitivity, linear dynamic range, and precision. High PCR efficiencies are typically a hallmark of robust assays, especially in relative quantification scenarios where calibrator C_t_s are directly compared to sample C_t_s (Pfaffl, 2001, 2006; Bustin et al., 2009). Efficiencies are calculated using calibration curves, consisting of six, 10-fold dilutions of target DNA (guidelines recommend at least five dilutions; Bustin et al., 2009). Logarithms of template concentration are plotted on the x-axis and C_t_s on the y-axis. LightCycler® 480 software fits a polynomial model to data to determine efficiency. Analytical sensitivity is validated by empirical measurement of the lower limit of target detection, known as LOD. The Clinical Laboratory Standards Institute (CSLI) defines LOD as “the lowest amount of analyte (measurand) in a sample that can be detected with (stated) probability, although perhaps not quantified as an exact value” (Forootan et al., 2017; Clinical Laboratory Standards Institute, 2020). Linear dynamic range describes the upper and lower limits of detection and quantification within acceptable thresholds of linearity and efficiency. Linearity of a standard curve is expressed as a correlation coefficient (*R*^2^). Minimum performance thresholds for all triplex assay calibration were set to *R*^2^ ≥ 0.98 (linearity) and 80–120% efficiency (Bustin et al., 2009). For quantification purposes assay efficiency is recommended to fall within a narrower, 90–110% range (Broeders et al., 2014). Material matrix type and DNA extraction methods can affect the dynamic range; thus, assay performance parameters are evaluated using target DNA extracted using two different methods, from two different matrices, at two different concentrations. Pea and soy DNA were extracted from dry pulse and protein powder, using both a CTAB and an SDS approach. Six-point, 10-fold dilution calibration curves using a high (~100 ng) and low (~25 ng) target amount were created. All dilutions were run in triplicate, for a total of 144 reactions for each pea and soy.

In addition to LOD sensitivity measurements, quantitative assays should include a limit of quantification (LOQ) sensitivity determination (Broeders et al., 2014). LOQ is defined by CSLI as “the lowest amount of measurand in a sample that can be quantitatively determined with (stated) acceptable precision and stated, acceptable accuracy, under stated experimental conditions” (Clinical Laboratory Standards Institute, 2020). No general guidelines about precision thresholds for LOQ determination exist; thus, a threshold must be selected that is appropriate for each unique test. Some literature proposes the lowest quantifiable concentration with a CV < 35% representing the LOQ, but more conservative guidelines suggest that CV ≤ 25% for quantitative assays (Broeders et al., 2014; Forootan et al., 2017).

Estimates of precision are expressed as SD or relative SD (RSD) values of reaction replicates. All reactions were run in triplicate as is recommended in guideline literature (Newmaster et al., 2019).

### Repeatability and Reproducibility

Repeatability of the assay is expressed as a percent agreement of true positive and negative results from samples analyzed over 2 days by the same operator on the same device. Validation guidelines recommend testing a minimum of seven targets and three non-targets so that sample sizes suffice for 95% confidence according to the simplified Cochran approach (Newmaster et al., 2019). All samples should be run in triplicate for a total of 60 reactions over 2 days. Reproducibility is assessed with the same protocol, except that the 2 days of testing should take place in two different labs or by two different operators. In this case, reproducibility was evaluated by two different operators.

### Mixture Testing

**Figure 1:**
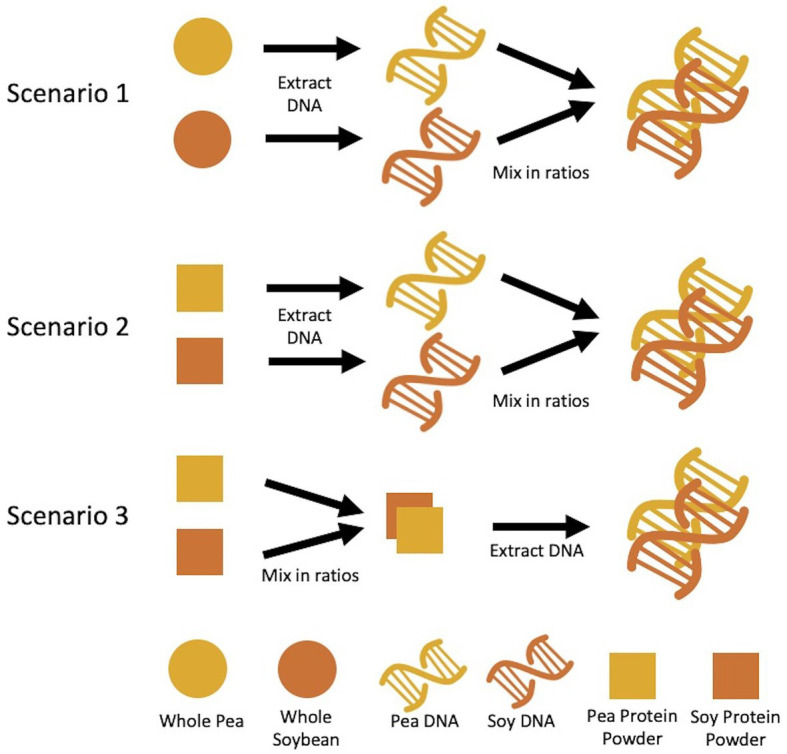
Methodology for mixture preparation of pea and rice materials. Scenario 1 and 2 describe DNA mass ratios created after extraction from starting materials, whereas scenario 3 described creation of ratios by starting material mass, followed by DNA extraction.

Three sets of mixtures were created based on the following mass ratios of pea to soy – 1:99, 5:95, 10:90, 25:75, 50:50, 75:25, 90:10, 95:5, and 99:1. The first set is based on DNA mass ratios (%wt/wt) using DNA extracted from raw pea and soy (dry pulse) material. The second is based on DNA mass ratios using the DNA extracted from pea and soy protein powders. The third set is based on dry weight mass ratios of the pea soy protein powder. The first two sets of mixtures involved first extracting DNA from pea and soy material separately, then mixing in DNA mass ratios. The third set of mixtures involved mixing protein powders in ratios by powder weight, then extracting DNA from the combined pea/soy material (Figure 1).

Mixture testing also required optimization of template concentration for reactions. Each set of fresh and processed DNA mass mixture ratios were tested for a total of 10, 5, and 1 ng. For example, a 50:50 pea to soy ratio by DNA mass would include 5 ng of pea and 5 ng of soy in a 10 ng reaction, 2.5 ng pea and 2.5 ng soy in a 5 ng reaction, and 0.5 ng of pea and 0.5 ng of soy in a 1 ng reaction. Calibrator DNA was not factored into the template total and a total mass of 0.026 ng calibrator was added into every reaction.

#### Data Analysis

Following collection of C_t_ value raw data, a relative quantification method was used to quantify soy and pea DNA in samples. This method involves comparison of sample C_t_ values to calibrator values. A delta-delta C_t_ efficiency model by Pfaffl (2001) was used to determine relative quantification of soy and pea:

$$\mathrm{Ratio}=\frac{{\left(E_{target}\right)}^{\Delta Cttarget\;\left(control-sample\right)}}{{\left(E_{calibrator}\right)}^{\Delta Ctcalibrator\;\left(control-sample\right)}}$$

Where,

E_target_ = Efficiency of target sample reaction

E_calibrator_ = Efficiency of the calibrator sample reaction

ΔCt_target_ = threshold cycle of target control – threshold cycle of the test sample

ΔCt_calibrator_ = threshold cycle of calibrator control – threshold of the test calibrator

To note, “treated” samples in our application were simply test samples of any proportion of target that would be compared to the 100% “control.”

### Fragment Size Analysis

An analysis of variance in fragment size of extractable DNA from soy and protein powders was conducted using an Agilent TapeStation® 4150 (Agilent, Santa Clara, CA), with samples run at the Genomics Advanced Analysis Centre at the University of Guelph, ON. A total of 10 soy protein powder samples were collected that were processed in 2019, 10 soy protein powder samples that were processed in 2017, and 13 pea protein powder samples that were processed in 2019. DNA was extracted, and 5 ng of genomic DNA from each sample was run on a TapeStation® DNA5000 ScreenTape® chip (measures fragment size distribution in the 100–5,000 bp range), with data were analyzed in TapeStation® Analysis Software 3.1 (Agilent, Santa Clara, CA). Data were assesed for normality and equality of variance using the Shapiro-Wilk test and the Levene’s test, respectively. Violation of equal variance prompted the use of the Welch’s *t*-test to assess differences in average fragment size between the 2019 and 2017 soy powder lots. Analysis was performed in the software R (R Core Team, 2020).

## Results and Discussion

### Primer and Probe Design for *Glycine max* (L.) Merr. and *Pisum sativum* L. Targets

The purpose of this study was to demonstrate the utility of a triplex assay for the detection and relative quantification of pea and soy botanical material. An important feature of successful assays is specific primers and probes that allow sufficient taxonomic resolution for identification of a target among non-target adulterants or closely related species (Newmaster et al., 2019). Design of suitable genetic markers for both soy and pea were confined within the bounds of several of the most documented and researched “barcode” regions for plants (see Materials and Methods). Using these regions holds an advantage in practice since more comprehensive libraries of certified reference sequences exist for more taxa than for alternative areas of the nuclear and plastid genomes. As time goes on, and the number of full reference genomes increases, this argument will lose relevance. For the purpose of designing an assay for current implementation, typical barcode-markers will better serve industry partners who have started to develop sequence libraries for vouchered reference material libraries. Markers for both pea and soy had to be sufficiently divergent from homologous sequences of industry-relevant, potential adulterants (Newmaster et al., 2019). This assay is designed as a tool for identification of pea and soy protein powders, thus relevant non-target species include common plant-based protein powder sources. Similar to pea and soy, many of these sources come from the *Fabaceae* family; thus, at minimum, generic level resolution is required to distinguish targets from non-targets (Supplementary Table S1). In this case, congeneric species are not common adulterants.

In the case of *G. max*, the nuclear ITS2 exhibited sufficient taxonomic resolution, while providing appropriate sequence options to design a primer and probe set that satisfied size criteria and thermodynamic requirements. Primers were designed that flanked a suitable 81 bp region. Since this qPCR molecular authentication pipeline does not include sequencing, a caveat is that unique SNPs must occur within the probe annealing region of the amplicon for the probe to differentiate the target from closely related species with similar homologs (Life Technologies, 2014; Newmaster et al., 2019). Specificity of primers is also important in order to exclusively allow amplification of targets. Oligos for *P. sativum* were designed within the chloroplastic *accD*, with suitable primers flanking a 131 bp region of the gene. Evaluation of thermodynamic criteria is summarized in Supplementary Table S3, detailing likelihood of secondary structure formation within each oligo and between each pair among the nine oligos that make up the triplex assay. Risk of interactions was deemed permissible for the assay according to this analysis.

#### ITS2 and *accD* Congeneric Resolution

Finding an appropriate region for taxonomic discrimination in plant models routinely comes with challenges, due to clade-specific success with some markers and lack of a universal barcode akin to CO1 for animals (Kress et al., 2005; Hollingsworth et al., 2011). When selecting appropriate targets for an assay, superior specificity is the goal, but minimal required resolution is determined by the relevant non-targets to the application. Congeneric amplification may be permissible in some cases where discrimination from more distantly related species is the focus. In this application, the main concern lay in differentiation of targets from non-targets that were, at closest, confamilial (*Fabaceae*).

Superior resolution has been documented for nuclear ITS2 in some medicinal plants; here, it appeared a suitable marker for *G. max* (Chen et al., 2010; Gao et al., 2017). The International Legume Database & Information Service (ILDIS) includes 162 names within the genus *Glycine*, most synonymous with others for a total of 14 or 15 accepted species, based on designation of domestic and wild soybean (Roskov et al., 2006). *Glycine* subgenus *Glycine* contains 13 species with low global coverage (mostly in Australia), whereas subgenus *Soja* contains the important, domesticated *G. max* (L.) Merr. (native to East Asia) and its wild relative *G. max subsp. soja* (Siebold & Zucc.) H.Ohashi, which is routinely treated in literature as a separate species: *Glycine soja* (Roskov et al., 2006). Whole genome sequencing studies revealed only 2.35% sequence difference between *G. max* (L.) Merr. and *Glycine soja* (Kim et al., 2010). The 81 bp ITS2 amplicon did not differ in *G. max* (L.) Merr. and *G. soja*, but other congeneric species did diverge (according to BLAST results in Genbank). *Glycine tomentella* Hayata had a minimum 2.74% sequence divergence from *G. max* (L.) Merr., and *Glycine microphylla* Tindale, a 4.05% divergence.

*Pisum* is a smaller genus native to southwest Asia and northeast Africa, with only three species accepted by ILDIS (one with two subspecies). *Pisum sativum* L. is the cultivated field pea that is a major human food crop. The 131 bp region of *accD* did not differentiate *Pisum fulvum* Sibth. & Sm. from *P. sativum* L. but had a 0.76% divergence in between *Pisum abyssinicum* A. Braun and *P. sativum* L. Interestingly, *accD* emerged as one of the few regions with sufficient variability to discriminate among closely related species. Previous work has documented high variability due to insertions of tandem repeats and has linked the region to nuclear-cytoplasmic conflict in the wild and domesticated pea (Nováková et al., 2019). Additionally, fragmentation and deletion of this region has been documented in several plant plastid genomes, with *accD* being completely deleted in the maize and wheat plastomes, and partially in rice (Maier et al., 1995; Harris et al., 2013).

### Analytical Specificity

**Figure 2:**
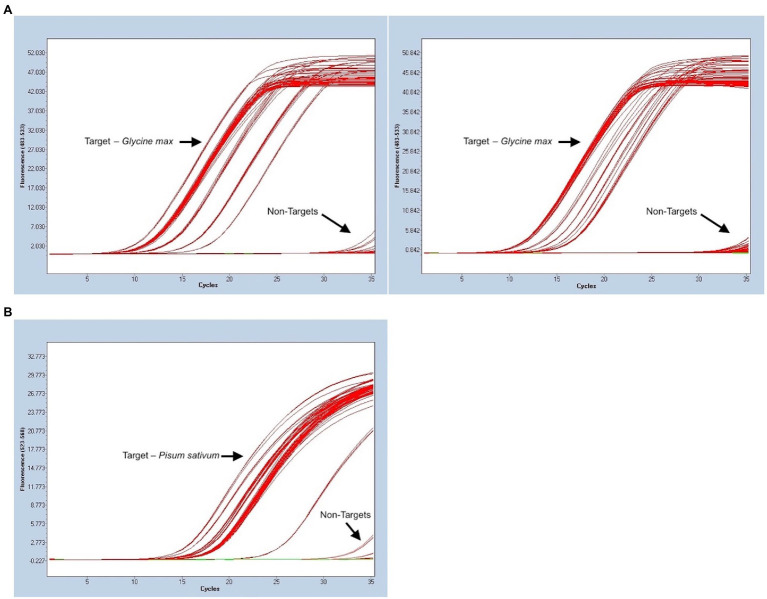
Evaluation of analytical specificity for both *Glycine max* (L.) Merr. (soy) and *Pisum sativum* L. (pea) portions of the triplex assay using a variety of sample matrices. C_t_ values are noted on the x-axis and fluorescent signal on the y-axis. **(A)** 33 target samples of soy and 13 non-targets, all run in triplicate. Two separate runs were complete since all samples could not fit in 96-well PCR plate. **(B)** 21 target samples of pea and 13 non-targets, all run in triplicate. Three samples with a later signal that departs from other targets are positive targets from a matrix that rendered low DNA quantity in extraction.

**Figure 3:**
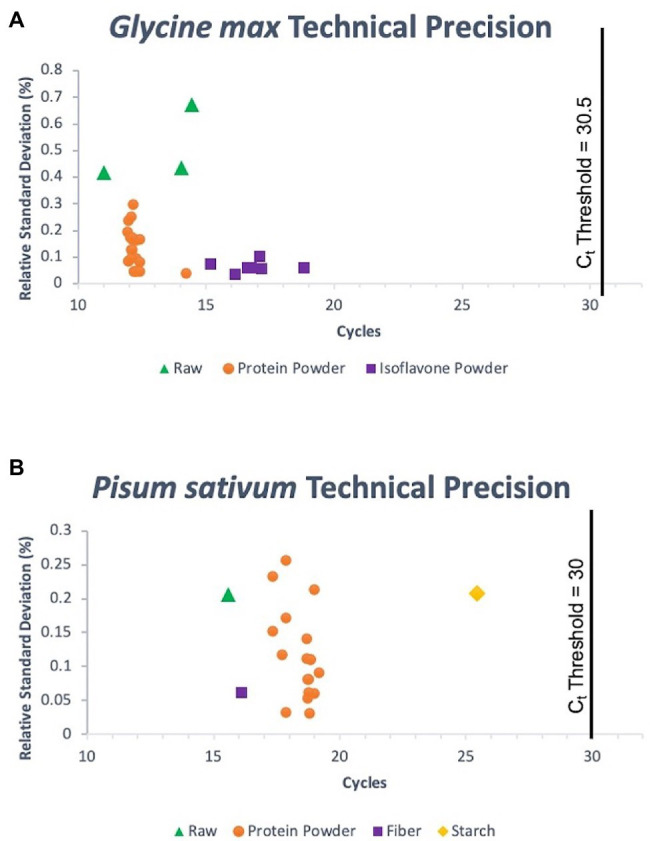
Technical precision for *G. max* (L.) Merr. **(A)** and *P.sativum* L. **(B)** triplicate reaction sets for all target samples run in the assessment of analytical specificity of the assay. C_t_ thresholds are denoted by vertical lines on each graph that represent the distinction between positive samples and off-target amplification.

The panel used to evaluate the specificity of pea and soy primer/probe sets performed as expected for a successful, specific assay. 33/33 (100%) true positive soy samples gave positive signals, and 13/13 (100%) of non-targets existed as negatives below a determined threshold, for both soy and pea samples (Figure 2). Consequently, this meant that the false positive and false negative rates for soy and pea assays were 0%. Concentration of DNA extraction elutes of true positive soy samples ranged from 0.66 to 59 ng/μl. Target sample C_t_s (average of triplicates) ranged from 11.02 to 18.83. Concentration of DNA extraction elutes of true positive pea samples ranged from “Out of Range – Low” (<0.01 ng/μl) to 33.8 ng/μl. Target sample C_t_s ranged from 15.57 to 25.44. Figure 3 summarizes the range of C_t_ values for each target matrix and shows the low RSD of each target sample’s set of triplicate reactions. Soy sample replicates had an RSD ≤ 0.673%, and pea samples an RSD of ≤0.257%, suggesting exceptional technical precision.

BLAST searches and other *in silico* tools for specificity screening of potential oligos for an assay are useful, but empirical testing for specificity with true positive samples and a vouchered non-target reference library is always required (Hübner et al., 2001; Bustin et al., 2009). A common occurrence in some assays is positive amplification curves being visible at very late C_t_ values for non-targets (Newmaster et al., 2019). This issue may be addressed by setting a C_t_ threshold, where any apparent positive curves with a later C_t_ value are disregarded as off target-amplification. This only works if there is a considerable gap between the earliest non-target C_t_s at a given concentration and the latest target C_t_s that are part of the intended linear dynamic range for the purpose of the assay. In our laboratory, we use the general rule that a threshold C_t_ should represent a minimum of one order of magnitude away from off target signals, based on back calculated concentrations. One 10-fold dilution of an assay with an efficiency close to 100% translates to a ~3.3 cycle difference (Life Technologies, 2014). Non-target C_t_s were tested in triplicate at 5 ng DNA and were ≥33.8 in the FAM fluorescence channel and ≥33.5 in the HEX channel. A C_t_ threshold of 30.5 cycles was determined for soy and a threshold of 30 cycles for pea. Any apparent positive amplification curves that come after these values are to be disregarded. These cutoffs were sufficiently far from non-targets and still supported the intended linear dynamic range of the assay. To note, no off-target amplification was observed in the Cy5 fluorescent channel, corresponding to the calibrator portion of the assay.

### Assay Performance

Efficiency calculations were performed for eight different standard curves for both pea and soy (all comprised of six, 10-fold dilutions). Both assays exhibited exceptional efficiency for both extraction methods (CTAB and SDS) and both matrices (fresh and processed), at high and low starting concentrations. Efficiencies were as follows: Soy Fresh CTAB high/low – 106.5/98.1%, Soy Fresh SDS high/low – 100.8/102.3%, Soy Processed CTAB high/low – 96.3/97.3%, Soy Processed SDS high/low – 97.4/98.9%, Pea Fresh CTAB high/low – 93/99.2%, Pea Fresh SDS high/low – 104.2/99.9%, Pea Processed CTAB high/low – 99.2/99.9%, and Pea Processed SDS high/low – 102.3/103.8%. Efficiency calculations are summarized in Supplementary Table S4 for CTAB-extracted, fresh soy DNA at high and low starting quantities, and in Supplementary Table S5 for CTAB-extracted, fresh pea DNA at high and low starting quantities. Efficiency calculations are summarized in Supplementary Table S6 for CTAB-extracted, processed soy DNA at high and low starting quantities, and in Supplementary Table S7 for CTAB-extracted, processed pea DNA at high and low starting quantities.

Analytical sensitivity, expressed as the LOD, is typically identified as the lowest concentration that is detectable with reasonable certainty (typically 95% confidence; Newmaster et al., 2019). Both pea and soy assays displayed 100% positive amplification in all replicates at the calibration curves’ lowest concentrations (with low RSD). This would suggest LODs as low as 0.15 pg for processed pea protein powder, 0.25 pg for fresh pea, 0.25 pg for processed soy, and 0.25 pg for fresh soy. RSDs were low; thus, these values could be also be acceptable as the assay LOQs. However, comprehensive optimization of the triplex assay involves setting a sensitivity threshold if late C_t_ amplification is observed for any non-targets (as in this case for pea and soy). The C_t_ cutoff of 30 for pea and 30.5 for soy restricts the linear dynamic range of the assay. Using series of three 2-fold dilutions, we determined the smallest amount of target DNA template that resulted in C_t_ values just before the determined threshold. With this strategy, the LOD/LOQ of the soy assay was determined to be 2.5 pg for fresh material, and 0.625 pg for processed protein powder. The LOD/LOQ of the pea assay was determined to be 1.25 pg for fresh material and 7.5 pg for processed protein powder. Thus, the experimentally validated linear dynamic ranges for each assay were 100–0.0025 ng and 100–0.000625 ng for fresh and processed soy material, respectively, and 100–0.00125 ng and 100–0.0075 ng for fresh and processed pea material, respectively.

Precision is expressed as SD and calculated over a minimum of three technical replicates. In order to discriminate a 2-fold change of target concentration with a 95% CI, an SD of ≤0.250 cycles must be observed, and an SD of ≤0.167 cycles to discriminate with a 99.7% CI (Applied Biosystems, 2016; Zhao et al., 2016). Exceptional precision was observed for fresh soy (SD = 0.139 cycles), processed soy (SD = 0.0321 cycles), fresh pea, (SD = 0.0451 cycles), and processed pea (SD = 0.0833 cycles).

### Repeatability and Reproducibility

Both soy and pea assay portions showed good repeatability and reproducibility. About 100% of the 42 target material reactions, conducted over 2 days by the same operator revealed positive amplification curves for both pea and soy. About 100% of the 18 non-target reactions were negative (no amplification or late curves after the predetermined C_t_ specificity threshold) for both soy and pea. Reproducibility tests over 2 days with two different operators revealed the same results.

### Mixture Testing

The goal of this study was to investigate the utility of a multiplex qPCR assay for mixtures of processed protein powders. The first challenge was to design an assay with appropriate specificity, sensitivity, efficiency, and precision that could retain superior analytical capability with degraded DNA template. Following our successful design and evaluation of assay performance using only one target at a time as template, came the challenge of testing multiplex capability.

The total quantity of template (pea and soy DNA targets) in reactions can affect quantitative estimates and overall quantitative linearity (the agreement between expected values and assay measurements) and must be optimized. Bulk qPCR reactions can be influence by amplification bias within a reaction, based on the thermodynamic properties of the assay components and spatial constraints of the reaction (Zhao et al., 2016). For example, a target that is in very low concentration compared to other targets in the multiplex reaction may experience a decrease in amplification efficiency due to reduced probability of stochastically binding primers and probe together with polymerase in the target sequence (Zhao et al., 2016). Upon testing three different target quantities (1, 5, and 10 ng) the optimal amount of total template DNA was found to be 5 ng.

All mixtures were quantified using the Pfaffl method for relative quantification, with efficiency correction capability (Pfaffl, 2001). Without efficiency correction, equations assume a 100% assay efficiency (i.e., a perfect doubling of targets every cycle), which may not reflect reality. Including efficiency correction provides more reliable and accurate quantitative estimations, hence the selection of the Pfaffl method of data analysis (Pfaffl, 2006). Pfaffl (2006) discussed that small difference in qPCR efficiency (∆E) of 3% (∆E = 0.03) between a low copy target sequence and a medium copy calibrator would result in an incorrectly calculated difference of expression ratio of 242% (E_target_ > E_calibrator_) after 30 PCR cycles. Greater differences in efficiency would result in more dramatic discrepancies – ∆E = 0.05 (432% gap), ∆E = 0.10 (1744% gap) – highlighting the advantage of efficiency correction (Pfaffl, 2006).

#### Quantification of Fresh, Pulse-Derived DNA Mixtures

**Figure 4:**
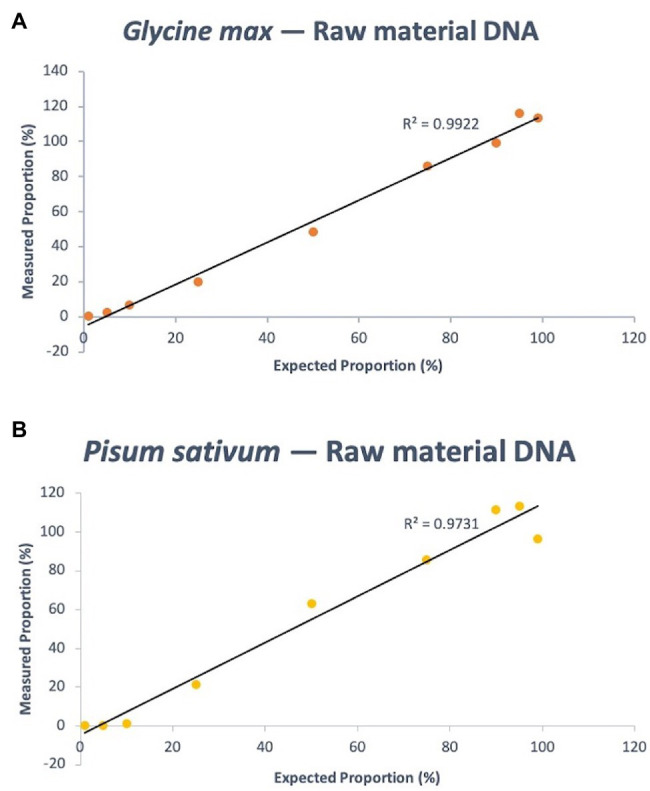
Quantitative linearity of the soy **(A)** and pea **(B)** portions of the triplex assay using DNA extracted from fresh materials (dry pulse). Agreement between expected proportions and measured proportions is plotted for expected DNA mass proportions of 99, 95, 90, 75, 50, 25, 10, 5, and 1%.

Eleven mass ratios of soy and pea DNA were tested using the assay, equating to single ingredient proportion estimates of 100, 99, 95, 90, 75, 50, 25, 10, 5, 1, and 0% (note that 100 and 0% values served as controls off of which other values were calculated, using the Pfaffl method). The expected composition of soy DNA in the mixtures – 99, 95, 90, 75, 50, 25, 10, 5, and 1% – were calculated using the C_t_ output of the PCR run to be 113.23, 115.93, 99.23, 86.23, 48.29, 20.00, 6.69, 2.37, and 0.22%, respectively. The quantitative linearity of the assay using fresh, pulse-derived soy DNA had a coefficient of variation (*R*^2^) of 0.9922 (*p* < 0.0001; Figure 4). For pea, the same known percent compositions were determined *via* qPCR as 96.56, 113.39, 111.50, 85.84, 63.33, 21.38, 0.96, 0.049, and 6.82E-05%, respectively. *R*^2^ of the linear regression analysis of known pea concentration vs. measurement agreement was 0.9731 (*p* < 0.0001; Figure 4). Supplementary Tables S8 and S9 shows the raw data (C_t_ values) of soy and pea targets and calibrator that were used to calculate quantity estimates using the Pfaffl equation for relative quantification with efficiency correction (see Materials and Methods for equation).

#### Quantification of Processed, Protein Powder-Derived DNA Mixtures

**Figure 5:**
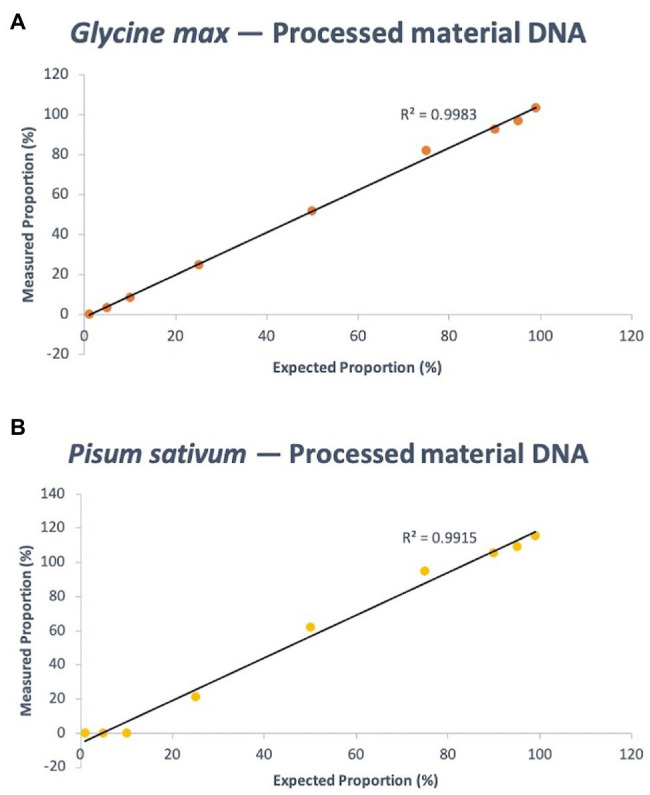
Quantitative linearity of the soy **(A)** and pea **(B)** portions of the triplex assay using DNA extracted from processed materials (protein powder). Agreement between expected proportions and measured proportions is plotted for expected DNA mass proportions of 99, 95, 90, 75, 50, 25, 10, 5, and 1%.

The same experimental design as the fresh-derived DNA mixtures was used for the protein powder derived DNA mixtures. The known composition of soy DNA in the mixtures – 99, 95, 90, 75, 50, 25, 10, 5, and 1% – were calculated using the C_t_ output of the PCR to be 103.62, 96.82, 92.76, 82.20, 51.95, 24.75, 8.47, 3.27, and 0.20%, respectively. This resulted in a quantitative linearity *R*^2^ of 0.9983 (*p* < 0.0001). For pea, the same known percent compositions were determined *via* qPCR as 115.91, 109.30, 105.65, 95.05, 62.44, 21.55, 0.30, 0.029, and 1.58157E-05%, respectively. *R*^2^ of the linear regression analysis of known pea concentration vs. measurement agreement was 0.9915 (*p* < 0.0001; Figure 5). Supplementary Tables S10 and S11 shows the raw data (C_t_ values) of soy and pea targets and calibrator that were used to calculate quantity estimates using the Pfaffl equation.

The exceptional linearity and minimal bias for the estimated percent composition values of pea and soy from the assay serves as a proof of concept for the utility of quantitative assays in processed protein powder mixtures. The similarity of the linearity among raw materials and processed materials is an encouraging result that suggests versatility of the assay with different matrices. This is not always the case, as matrix effects can have significant influence on key PCR parameters like efficiency (Cankar et al., 2006). Inhibitory or faciliatory compounds in the DNA elute following extraction may have a different composition in fresh material as opposed to processed, underlining the importance of assay validation for each unique matrix. It is also worth noting that, while both target assays produced similar linearity, the raw soy assay measurements had greater accuracy than the pea. The difference in the estimated percent DNA proportion vs. expected proportions for soy was at most 7.2%, with an average of 2.5%; and for pea was at most 20%, with an average of 10.9%. However, the high *R*^2^ suggests a strong relationship that can be used to make an algorithmic correction for pea measurements. More replication will further elucidate the stability of this relationship.

#### Quantification of Protein Powder Dry-Mass Mixtures

The final set of mixture ratios tested using the assay were mass ratios based on dry weight of protein powders. A unique challenge presented itself with correlation of quantitative estimates from the assay with expected proportions of powder. The assay is optimized for a total template quantity of 5 ng and estimates the proportion of target soy or pea DNA by comparison to its corresponding 100% control. Soy and pea protein powder were empirically determined to render different quantities of DNA in an extraction with the same parameters. Soy (*n* = 5) rendered an average DNA concentration (60 μl elution) of 20 ng/μl ± 0.757 (±SD) and pea (*n* = 5) rendered an average of 3 ng/μl ± 0.436. The difference in extractable DNA quality from the same quantity of protein powder between the two sources meant that expected proportions of sources based on molecular weight of DNA needed to be adjusted to reflect protein powder ratios. Thus, proportions of 99, 95, 90, 75, 50, 25, 10, 5, and 1% protein powder dry mass, were reweighted for soy to be expected DNA mass proportions of 99.85, 99.22, 98.36, 95.24 86.95, 68.97, 42.55, 25.97, and 6.31%, respectively; and for pea to be 93.69, 74.03, 57.45, 31.03, 13.04, 4.76, 1.639, 0.78, and 0.15%, respectively. The lower percent compositions for pea had to be adjusted downward, but even the smallest quantity (0.15% of 5 ng total template = 7.5 pg) still falls within the dynamic range supported by the assay. Soy proportions were measured by the assay (in order of decreasing expected percent) to be 95.51, 70.82, 55.55, 25.13, 15.66, 9.40, 3.78, 1.17, and 0.166%. These results were non-linear but exhibited a pattern that could be modeled by an exponential function (*R*^2^ = 0.9541; *p* < 0.0001; Supplementary Figure S1). Quantitative results suggested an underrepresentation of soy DNA in several mixtures, with an increasing percent negative bias as the proportion of pea increases. Replication of the procedure with a higher amount of template revealed a similar relationship. Pea results also did not reflect a linear relationship (polynomial fit *R*^2^ = 0.9781; *p* < 0.0001), with general overestimation of proportions (estimates in order of decreasing proportions: 169.04, 156.38, 194.16, 335.43, 243.16, 134.08 30.21, 1.05, and 0.005394%; Supplementary Figure S1). To note, estimations theoretically should not exceed 100%, thus there may have been an issue with low pea template in the 100% control, or high template in the sample.

There are several possible explanations for the obfuscation of quantitative estimates that may involve sample preparation, upstream of PCR. Given the exceptional quantitative linearity of the assay with engineered DNA mass mixtures, that relationship should have been reflected with the adjusted DNA mass proportions of the protein powder mixtures. One potential introduction of bias involved a difference in the physical dynamics of the extraction procedure. During extraction, we observed the pea protein powder to absorb the lysis buffer more readily than the soy protein powder, with soy-dominant mixtures appearing more liquid than pea-dominant solutions. If exposure to lysis buffer was biased toward the more absorbent pea powder, further collection of lysate and DNA extraction would inflate the presence of pea DNA in the mixture. This would be consistent with the observed underestimation of soy presence, and overestimation of pea presence in quantitative results. This introduction of bias when mixtures are extracted underlines the importance of optimization of all steps in a molecular authentication and quantification pipeline. Further work should manipulate lysis buffer volumes and mass of powder used in extraction to find optimums. Several biological replicate mixtures should be created and assessed with the assay for linearity of quantitative estimates. If linearity does not improve but is consistent within an acceptable range or polynomial relationship, an algorithmic correction can be applied to achieve quantification.

### Fragment Size Analysis and Proposition of Standard Evaluation

Average fragment size within a range of 100–5,000 bp was measured for two groups of 10 soy protein powder samples, and 13 pea protein powder samples. The first goal of this analysis was to estimate the variability of template quality lot-to-lot, among samples that were processed in the same year, with the same protocol. The group of soy protein lots that were processed in 2019 (*n* = 10) had an average fragment size of 1,071 ± 44.287 bp (±SD) and the soy lots that were processed in 2017 (*n* = 10) had an average fragment size of 1166.3 ± 70.528 bp (±SD). The lots of pea protein powder (*n* = 13), that were all processed in 2019, had an average fragment size of 1807.2 ± 126.59 bp (±SD).

Pairwise comparison of the 2017 and 2019 soy protein powder group means identified a significant difference in average fragment length (*p* < 0.05; Supplementary Figure S2). This was an unexpected result, seeing as we predicted an equal or slightly lower average fragment size in the lots processed in 2017 as compared to 2019, driven by a gradual degradation of DNA in stored material. Possible explanations involve different storage practices or processing parameters. We procured material from a supplement company who purchased the material from a protein powder manufacturer. The understanding was that the 2017 and 2019 lots of soy protein were processed using the same protocol. However, it is not common practice for raw material processing firms to notify downstream product manufacturers of minor procedure changes, so long as quality specifications remain constant (typically chemical measurements). Thus, even minor changes to volume, temperature, or time parameters during protein isolation could have resulted in the difference in average fragment size. Previous work has demonstrated the impact that several different processing steps could have on DNA quality and quantity, thus differences in DNA fragment size can be attributed to alterations in processing parameters (Faller et al., 2019). Influence from storage was unlikely, seeing as the lots were traceable – 2017 lots were immediately sampled into opaque bags and stored in a room temperature warehouse, and 2019 lots were sampled the same way and soon tested by us.

Regardless of the mechanism behind the difference in average fragment size between the two groups, implications of such variations include potential impact on the reproducibility of quantitative estimations. Reliable relative quantification requires representative standards or at least an understanding of all relevant standard parameters. Unknown samples that differ in average fragment size compared to the standard can result in obfuscation of quantification, depending on the magnitude of deviation from the standard. However, if an estimation of average fragment size is conducted on unknown material before analysis using the assay, the appropriateness of the standard can be assessed, and results can be algorithmically adjusted to reflect an accurate comparison to the standard. This can be a very simple process, centered around observation of amplification success of different sized genomic targets, using a SYBR green, real-time PCR approach (similar to a model our lab has previously used – testing PCR success with 100, 200, and 300 bp targets for processed green tea extract; Faller et al., 2019). Estimation of average fragment size can be determined for both the standard and unknown, and any difference that falls within an acceptable range can serve as evidence that the assay can be used with the given unknown. The tolerable level of deviation in fragment size between an unknown and a standard depends on the required precision of the assay and must first be empirically tested with replicate standard curves. With this approach, a threshold in deviation can be identified that will judge an unknown compatible with the standard or requiring of an algorithmic correction post-PCR. Other methods have also been explored. Brisco et al. (2010) created a protocol by which DNA integrity could be measured and incorporated into qPCR assay data analysis. They assume that lesions in DNA (caused by degradative processes) occur randomly and can be described by the Poisson distribution. Their goal was to create a model simple enough to enable use in routine PCR, intended for application in quantification of minimal residual disease in human leukemia. In that application, degraded DNA also presents a challenge of judging what fraction of extracted target DNA is actually amplifiable by PCR. They determined that the integrity of a DNA sample (*r* – mean number of lesions per base in the sequence) can be modeled using PCR efficiency (*a*) and the slope of the linear relationship between C_t_ and the length of the amplicon (Equation: *r* = slope · log_e_a). The determined amplifiable fraction of target DNA in unknowns compared to a reference can then be used to decide upon the data analysis pipeline (Brisco et al., 2010).

Another important metric to comment on is the SD among lots of protein powder that were verified to be processed by the same procedure, in the same relative time frame. The relative SD of each group reflects the clustering of data around the mean. The low RSD of the 2017 soy lots, 2019 soy lots, and 2019 pea lots – 6.05, 4.14, and 7.00% – suggest tight clustering around the mean and limited variation in fragment size. This provides important evidence for the reliability and reproducibility of the assay used with different lots of material, processed temporally close together. Importantly, this reflects the rapid testing pipeline of companies in industry, who receive many lots of material from the same manufacturer, daily.

### Challenges and Implementation

There is an increasing demand for orthogonal analytical techniques that can form comprehensive quality control pipelines to address all relevant analytes for product authenticity and quality verification (AOAC – AOAC International; Guideline Working Group, 2012). Having cheap methods with high throughput that can act as an initial screening tool for identity and proportionality of ingredients, offers a significant benefit to companies. The observed success in using this assay to discern percent composition of soy and pea DNA in mixtures suggests potential use for judging presence of a target as being a minor contaminant or significant adulterant. Identifying this threshold is dependent upon understanding the expected DNA quality and quantity that is extractable from a given amount of protein. This way, expected percent composition of target DNA in a mixture can be weighted based on its source, and PCR C_t_ values can be corrected based on DNA integrity of a sample. Though there are a multitude of botanical species that emerge as adulterants in commercial NHPs, targeted qPCR tools can be a cost-effective addition to quality control programs for routine screening of the most-common unintended ingredients. For example, another leguminous genus, *Lupinus* spp. (lupin), is used to make lupin flour, commonly used as a soybean protein substitute (Scarafoni et al., 2009). It is avoided due to allergen concerns in some products, and sensitive qPCR approaches have been designed for the specific purpose of detecting lupin in similar flour products (Scarafoni et al., 2009; Demmel et al., 2012). This tool is relevant and efficient in this context but may not need to be included in other products’ analytical testing programs. The assay in our study also demonstrates the use of targeted tools, and further highlights the informative additions of quantification and multiplex.

There are many sources of variation that can have influence on the quantity, quality, and amplifiable fraction of target DNA, many of which should be estimated for unknown samples, pre-PCR. Matrix effects in the context of DNA elutes can be described as the composition of secondary metabolites and grade of DNA integrity of a target, unique to a specific type of sample material (Rossen et al., 1992; Wilson, 1997; Holden et al., 2003; Cankar et al., 2006; Chapela et al., 2015). We carried out assay validation using fresh pulse and protein powder matrices that are distinct, even to the naked eye. However, different types of protein powder from different manufacturers can also be considered different matrices that will have their own matrix effects. We have discussed methods of judging DNA integrity, but secondary metabolites can also be evaluated by measurement with a spectrophotometer of 260/280 nm (A_260nm/280nm_) and 260/230 nm (A_260nm/230nm_) ratios. Differences in protein powder processing techniques of manufactures result in unique composition of secondary metabolites, which may influence PCR efficiency *via* enhancement or inhibition (Wilson, 1997; Cankar et al., 2006; Kubista et al., 2006). In this study, we used a synthetic calibrator, which controlled for variation among different reactions. Another avenue to explore is use of botanical calibrators that can be run through a DNA extraction process with sample material. This way, variation introduced during this process could also be controlled for.

Finding a suitable genetic marker for an assay requires appropriate taxonomic resolution, but other physical features of the sequences should be considered. Variation in copy number can exist based on sequence or sequence location. For example, ITS2 – the marker selected for discrimination of *G. max* (L.) Merr. from non-targets – is typically present in multiple copies in the plant genome, with variability in copy number being observed among different populations of a species (Rogers and Bendich, 1987; Hřibová et al., 2011). For pea, abundance of the chloroplastic *accD* marker in a volume of extracted genomic DNA can be variable, due to tissue-dependent abundance (Birky, 1983; Possingham and Lawrence, 1983). Additionally, differences in cell size and genome size in between targets will further complicate the association between measured powder weight or genomic DNA mass and the actual copy number of target sequences. For example, pea has a larger genome than soybean (Arumuganathan and Earle, 1991). All of these sources of variation underline the absolute necessity of empirical validation of the assay using representative standards. With an understanding of variation, and estimation of quality parameters where possible, qPCR can be a robust tool for the identification and quantification of different protein sources in a mixture.

## Conclusion

This study served as a successful proof of concept for the utility of qPCR assays in identification and quantification of protein powders. Performance was validated according to common guidelines, and the importance of evaluating DNA integrity for reliable standard creation was emphasized. It is important to understand that empirical validation is necessary for any unique matrix intended for use with the assay, and that stringent performance specifications should be met. Here, I developed a robust assay for the quantification of soy and pea target DNA in a mixture and discussed the necessary optimization for commercial implementation with protein powder mixtures. Methods need to be fit for purpose, and multiplex qPCR assays can achieve reliable identification of protein powders and quantification, given the required level of precision.

## Data Availability Statement

The original contributions presented in the study are included in the article/Supplementary Material, further inquiries can be directed to the corresponding author.

## Author Contributions

AF and SN conceived and designed the experiment. AF carried out all experimental validation work and analyzed the data. DS provided qPCR technical support throughout design. YZ, SR, and ZL provided validation design consultation. SR, YZ, ZL, PC, and GS helped procure botanical materials. PC, GS, and SN provided funding through respective organizations. All authors contributed to the article and approved the submitted version.

### Conflict of Interest

YZ, ZL, PC, and GS are employed by Herbalife International. The authors declare that this study received funding from Herbalife International. This funder had the following involvement in the study: provided botanical samples of soybean and pea raw materials, as well as design consultation for validation of the assay.
