# Supplementary material for: Validation of a Triplex Quantitative Polymerase Chain Reaction Assay for Detection and Quantification of Traditional Protein Sources, Pisum sativum L. and Glycine max (L.) Merr., in Protein Powder Mixtures
Source: Front Plant Sci. 2021 May 24;12:661770. doi: 10.3389/fpls.2021.661770 (PMC8183462; doi:10.3389/fpls.2021.661770)
Supplement: Supplementary file 1 [file Data_Sheet_1.docx]

**A)**


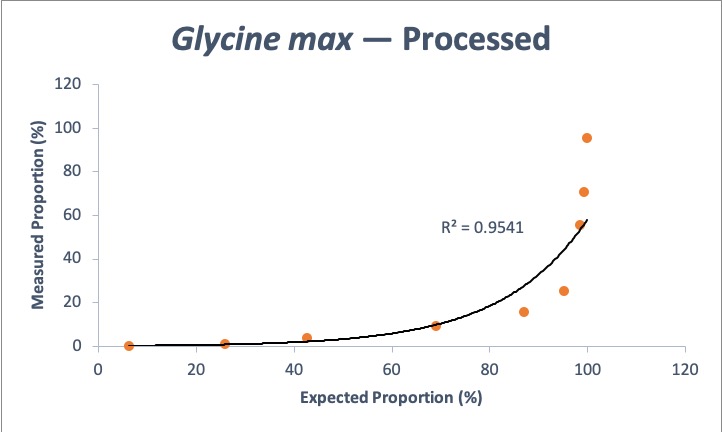


**B)**


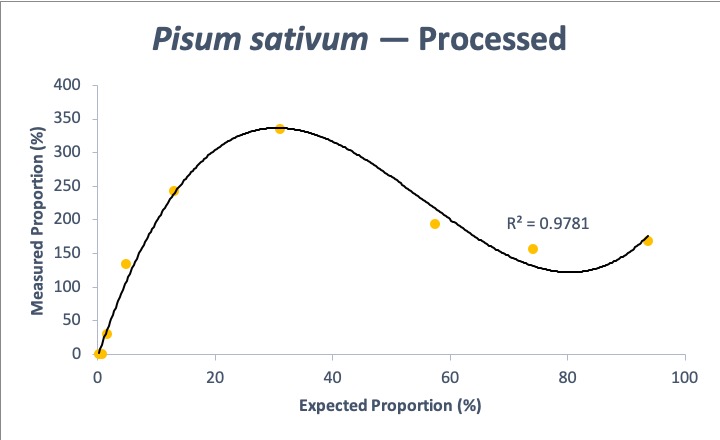


# Supplementary Figure S1: Quantitative linearity of the soy (A) and pea (B) portions of the triplex assay using DNA extracted from processed materials (protein powder). Ratios of mass mixtures were created using powders, and DNA was extracted as one elute. Thus, expected DNA mass proportions were adjusted. Agreement between expected proportions and measured proportions is plotted for expected DNA mass proportions of 99.85%, 99.22%, 98.36%, 95.24% 86.95%, 68.97%, 42.55%, 25.97% and 6.31% for soy and 93.69%, 74.03%, 57.45%, 31.03%, 13.04%, 4.76%, 1.639%, 0.78% and 0.15% for pea.


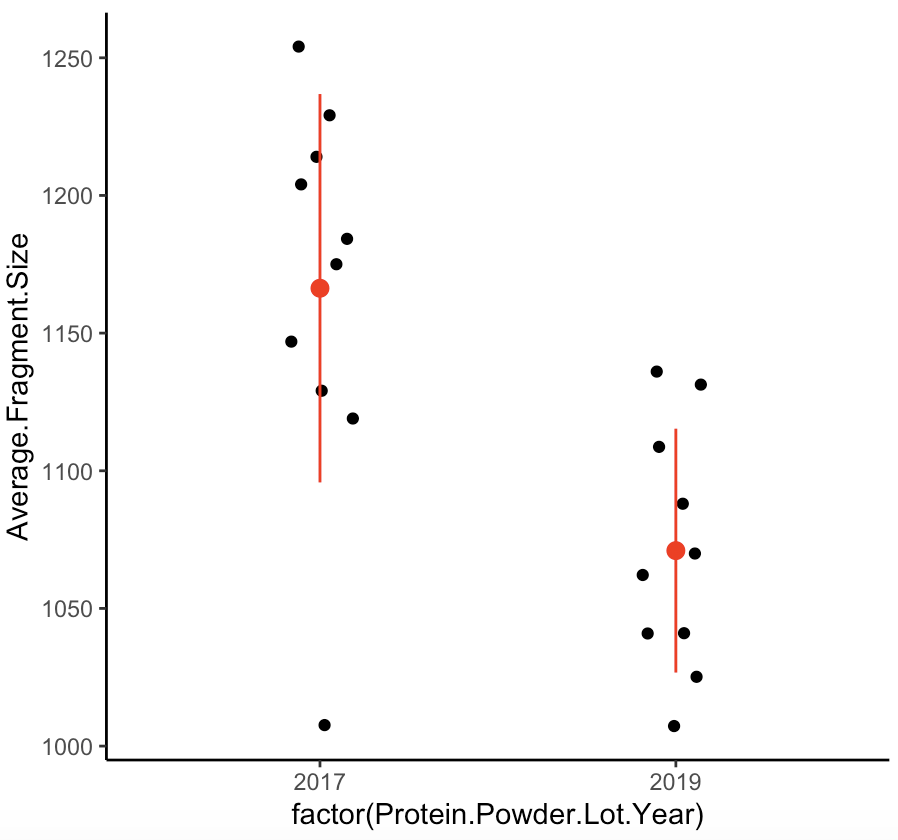


Protein Powder Lot Year

Average Fragment Size (bp)

*

**Supplementary Figure S2**: Comparison of soy protein powder lots processed in 2017 (n=10) and 2019 (n=10). The mean ± SD is plotted in red for each group of lots. A significant difference exists in between group means (Welch’s t-test, p<0.05).

**Supplementary Table S1:** Target and non-target materials used for assay design and analytical specificity evaluation (supplementary to vouchered herbarium references). This list includes commercial botanical samples and two organismal samples.

| **Latin Name** | **Family** | **Sample Designation** | **Composition/ Material Matrix** | **Sample Code** | **Batch Number** |
| --- | --- | --- | --- | --- | --- |
| *Glycine max* | Fabaceae | Target | Dry Whole Soybean | GM-HL-F1 | 063064 |
| *Glycine max* | Fabaceae | Target | Dry Whole Soybean | FC367 | 2019373295-RM346FC |
| *Glycine max* | Fabaceae | Target | Powder – Soy Protein Isolate 90% | 186BI | B701918 |
| *Glycine max* | Fabaceae | Target | Powder – 3% Soy Isoflavone | 1228 NW | 20115009 |
| *Glycine max* | Fabaceae | Target | Powder – 3% Soy Isoflavone | 360 NW | 20082822 |
| *Glycine max* | Fabaceae | Target | Powder – 3% Soy Isoflavone | 955 NW | 20105653 |
| *Glycine max* | Fabaceae | Target | Powder – 3% Soy Isoflavone | NW 53 | 20071795 |
| *Glycine max* | Fabaceae | Target | Powder – 3% Soy Isoflavone | 609 NW | 20092908 |
| *Glycine max* | Fabaceae | Target | Powder – 40% Soy Isoflavone | 194 NW | 20076618 |
| *Glycine max* | Fabaceae | Target | Powder – 40% Soy Isoflavone | 1177 NW | 20113554 |
| *Glycine max* | Fabaceae | Target | Powder – 40% Soy Isoflavone | 818 NW | 20100716 |
| *Glycine max* | Fabaceae | Target | Powder – Soy Protein Isolate | S4.1 | 062996 |
| *Glycine max* | Fabaceae | Target | Powder – Soy Protein Isolate | S4.2 | 062850 |
| *Glycine max* | Fabaceae | Target | Powder – Soy Protein Isolate | S4.3 | 062971 |
| *Glycine max* | Fabaceae | Target | Powder – Soy Protein Isolate | S4.4 | 063140 |
| *Glycine max* | Fabaceae | Target | Soy Protein Powder Isolate | S4.5 | 063203 |
| *Glycine max* | Fabaceae | Target | Powder – Soy Protein Isolate | S4.6 | 063206 |
| *Glycine max* | Fabaceae | Target | Powder – Soy Protein Isolate | S4.7 | 063259 |
| *Glycine max* | Fabaceae | Target | Powder – Soy Protein Isolate | S4.8 | 063261 |
| *Glycine max* | Fabaceae | Target | Powder – Soy Protein Isolate | S4.9 | 063262 |
| *Glycine max* | Fabaceae | Target | Powder – Soy Protein Isolate | S4.10 | 063260 |
| *Glycine max* | Fabaceae | Target | Powder – Soy Protein Isolate | S4.11 | 062885 |
| *Glycine max* | Fabaceae | Target | Powder – Soy Protein Isolate | S5.1 | 050011 |
| *Glycine max* | Fabaceae | Target | Powder – Soy Protein Isolate | S5.2 | 050012 |
| *Glycine max* | Fabaceae | Target | Powder – Soy Protein Isolate | S5.3 | 050231 |
| *Glycine max* | Fabaceae | Target | Powder – Soy Protein Isolate | S5.4 | 050288 |
| *Glycine max* | Fabaceae | Target | Powder – Soy Protein Isolate | S5.5 | 050295 |
| *Glycine max* | Fabaceae | Target | Powder – Soy Protein Isolate | S5.6 | 050296 |
| *Glycine max* | Fabaceae | Target | Powder – Soy Protein Isolate | S5.7 | 050304 |
| *Glycine max* | Fabaceae | Target | Powder – Soy Protein Isolate | S5.8 | 050305 |
| *Glycine max* | Fabaceae | Target | Powder – Soy Protein Isolate | S5.9 | 050329 |
| *Glycine max* | Fabaceae | Target | Powder – Soy Protein Isolate | S5.10 | 050330 |
| *Pisum sativum* | Fabaceae | Target | Dry Whole Pea | PS-HF-F1 | 000846-46 |
| *Pisum sativum* | Fabaceae | Target | Powder – Pea Fibre | AMW3 | R7570 |
| *Pisum sativum* | Fabaceae | Target | Powder – Pea Protein 84% | 933NW | 20105018 |
| *Pisum sativum* | Fabaceae | Target | Powder – Pea Protein 80% | 114BI | 17243 |
| *Pisum sativum* | Fabaceae | Target | Powder – Pea Starch | FC232 | 2228NVFCX10266 |
| *Pisum sativum* | Fabaceae | Target | Powder – Pea Protein 84% | 872NW | 20091687 |
| *Pisum sativum* | Fabaceae | Target | Powder – Pea Protein Isolate | P1.1 | 1039340 |
| *Pisum sativum* | Fabaceae | Target | Powder – Pea Protein Isolate | P1.2 | 1039341 |
| *Pisum sativum* | Fabaceae | Target | Powder – Pea Protein Isolate | P1.3 | 1039342 |
| *Pisum sativum* | Fabaceae | Target | Powder – Pea Protein Isolate | P1.4 | 1038252 |
| *Pisum sativum* | Fabaceae | Target | Powder – Pea Protein Isolate | P1.5 | 1038554 |
| *Pisum sativum* | Fabaceae | Target | Powder – Pea Protein Isolate | P1.6 | 1040792 |
| *Pisum sativum* | Fabaceae | Target | Powder – Pea Protein Isolate | P1.7 | 1042226 |
| *Pisum sativum* | Fabaceae | Target | Powder – Pea Protein Isolate | P1.8 | 1036689(AP) |
| *Pisum sativum* | Fabaceae | Target | Powder – Pea Protein Isolate | P1.9 | 1043515(AP) |
| *Pisum sativum* | Fabaceae | Target | Powder – Pea Protein Isolate | P1.10 | 1041263 |
| *Pisum sativum* | Fabaceae | Target | Powder – Pea Protein Isolate | P1.11 | 1042279 |
| *Pisum sativum* | Fabaceae | Target | Powder – Pea Protein Isolate | P1.12 | 1036688(AP) |
| *Pisum sativum* | Fabaceae | Target | Powder – Pea Protein Isolate | P1.13 | 1043514 |
| *Pisum sativum* | Fabaceae | Target | Powder – Pea Protein Isolate | P1.14 | 1036690(AP) |
| *Bos taurus* | Bovidae | Non-Target | Liquid – Milk | BTM1 | Cows #4316/#4634 (pooled) |
| *Acheta domesticus* | Gryllidae | Non-Target | Leg | AD1 | – |

**Supplementary Table S2:** Master mix for all reactions run during data acquisition.

| **Component** | **Stock Concentration** | **Volume (µL)** | **Final Concentration** |
| --- | --- | --- | --- |
| SensiFAST™ Probe No-ROX Master Mix | 2x | 15 | 1x |
| *G. max* ITS2 F | 10 µM | 1.5 | 500 nM |
| *G. max* ITS2 R | 10 µM | 1.5 | 500 nM |
| *G. max* ITS2 P | 10 µM | 0.75 | 250 nM |
| *P. sativum* accD F | 10 µM | 1.5 | 500 nM |
| *P. sativum* accD R | 10 µM | 1.5 | 500 nM |
| *P. sativum* accD P | 10 µM | 0.75 | 250 nM |
| Calibrator F | 10 µM | 0.6 | 200 nM |
| Calibrator R | 10 µM | 0.6 | 200 nM |
| Calibrator P | 10 µM | 0.3 | 100 nM |
| Calibrator DNA | 0.013 ng/µL | 2 | Quantity: 0.026 ng |
| Template | - | 4 | - |
| Total | - | 30 | - |

F = Forward Primer; R = Reverse primer; P = Probe

| **Oligo or Interacting Pair** | **2° Structure Evaluation** | **Maximum ∆G (Kcal/mol)** |
| --- | --- | --- |
| SF | Homodimer | -5.19 |
| SR | Homodimer | -6.76 |
| SP | Homodimer | -9.96 |
| PF | Homodimer | -12.9 |
| PR | Homodimer | -6.34 |
| PP | Homodimer | -3.61 |
| CF | Homodimer | -3.07 |
| CR | Homodimer | -4.85 |
| CP | Homodimer | -3.3 |
| SF-SR | Heterodimer | -5.19 |
| SF-SP | Heterodimer | -6.91 |
| SR-SP | Heterodimer | -6.76 |
| PF-PR | Heterodimer | -4.64 |
| PF-PP | Heterodimer | -10.05 |
| PR-PP | Heterodimer | -5.86 |
| CF-CR | Heterodimer | -3.07 |
| CF-CP | Heterodimer | -4.41 |
| CR-CP | Heterodimer | -3.07 |
| PF-SF | Heterodimer | -6.68 |
| PF-SP | Heterodimer | -3.61 |
| PF-SR | Heterodimer | -5.99 |
| PP-SF | Heterodimer | -6.75 |
| PP-SP | Heterodimer | -8.09 |
| PP-SR | Heterodimer | -5.02 |
| PR-SF | Heterodimer | -4.38 |
| PR-SP | Heterodimer | -5.02 |
| PR-SR | Heterodimer | -6.6 |
| CF-SF | Heterodimer | -4.52 |
| CF-SP | Heterodimer | -5 |
| CF-SR | Heterodimer | -5.13 |
| CF-PF | Heterodimer | -5.99 |
| CF-PP | Heterodimer | -4.64 |
| CF-PR | Heterodimer | -4.64 |
| CP-SF | Heterodimer | -6.21 |
| CP-SP | Heterodimer | -6.6 |
| CP-SR | Heterodimer | -5 |
| CP-PF | Heterodimer | -4.03 |
| CP-PP | Heterodimer | -6.7 |
| CP-PR | Heterodimer | -4.39 |
| CR-SF | Heterodimer | -3.89 |
| CR-SP | Heterodimer | -5 |
| CR-SR | Heterodimer | -6.5 |
| CR-PF | Heterodimer | -4.64 |
| CR-PP | Heterodimer | -7.96 |
| CR-PR | Heterodimer | -6.6 |

**Supplementary Table S3**: Summary of thermodynamic parameters (likelihood of secondary structure formation). Oligos were designed to keep interactions ∆G ≤ -9.0 Kcal/mole. If lower ∆G was unavoidable, interactions were judged permissible if dimers were not 3’ extendable.

SF – Soy Forward (Primer); SR – Soy Reverse; SP – Soy Probe; PF – Pea Forward; PR – Pea Reverse; PP – Pea Probe; CF – Calibrator Forward; CR – Calibrator Reverse; CP – Calibrator Probe

**Supplementary Table S4**: DNA dilutions and corresponding C_t_s for efficiency and linearity calculations. Extractions were complete using the CTAB protocol. A) Fresh soy DNA, high starting quantity. B) Fresh soy DNA, low starting quantity.

**A)**

| **Sample** | **DNA Template Quantity (ng)** | **Dilution** | **Ct values** | | | **Avg** | **SD** | **Efficiency** | **R^2^** |
| --- | --- | --- | --- | --- | --- | --- | --- | --- | --- |
|  |  |  | **Rep1** | **Rep2** | **Rep3** |  |  |  |  |
| GM-HL-F1 Whole Soybean | 100 | 10^0^ | 13.03 | 13.22 | 13.13 | 13.13 | 0.095 | 106.5% | 0.9998 |
|  | 10 | 10^-1^ | 16.37 | 16.31 | 16.12 | 16.27 | 0.13 |  |  |
|  | 1 | 10^-2^ | 19.72 | 19.76 | 19.71 | 19.73 | 0.026 |  |  |
|  | 0.1 | 10^-3^ | 23.38 | 23.33 | 23.35 | 23.35 | 0.025 |  |  |
|  | 0.01 | 10^-4^ | 27.54 | 27.38 | 27.31 | 27.41 | 0.12 |  |  |
|  | 0.001 | 10^-5^ | 31.15 | 31.18 | 31.34 | 31.22 | 0.10 |  |  |

**B)**

| **Sample** | **DNA Template Quantity (ng)** | **Dilution** | **Ct values** | | | **Avg** | **SD** | **Efficiency** | | **R^2^** |
| --- | --- | --- | --- | --- | --- | --- | --- | --- | --- | --- |
|  |  |  | **Rep1** | **Rep2** | **Rep3** |  |  |  |  |  |
| GM-HL-F1 Whole Soybean | 25 | 10^0^ | 15.41 | 15.31 | 15.31 | 15.34 | 0.058 | 98.1% | 89.3% | 0.9993 |
|  | 2.5 | 10^-1^ | 18.68 | 18.72 | 18.63 | 18.68 | 0.045 |  |  |  |
|  | 0.25 | 10^-2^ | 22.07 | 21.99 | 22 | 22.02 | 0.044 |  |  |  |
|  | 0.025 | 10^-3^ | 25.91 | 25.9 | 25.86 | 25.89 | 0.026 |  |  |  |
|  | 0.0025 | 10^-4^ | 29.75 | 29.64 | 29.71 | 29.7 | 0.056 |  |  |  |
|  | 0.00025 | 10^-5^ | 33.24 | 32.92 | 32.82 | 32.99 | 0.22 |  |  |  |

Avg = Average

SD = Standard Deviation

**Supplementary Table S5**: DNA dilutions and corresponding C_t_s for efficiency and linearity calculations. Extractions were complete using the CTAB protocol. A) Fresh pea DNA, high starting quantity. B) Fresh pea DNA, low starting quantity.

**A)**

| **Sample** | **DNA Template Quantity (ng)** | **Dilution** | **Ct values** | | | **Avg** | **SD** | **Efficiency** | **R^2^** |
| --- | --- | --- | --- | --- | --- | --- | --- | --- | --- |
|  |  |  | **Rep1** | **Rep2** | **Rep3** |  |  |  |  |
| PS-HL-F1 Whole Pea | 114 | 10^0^ | 14.76 | 14.76 | 14.75 | 14.76 | 0.0058 | 93.0% | 0.9999 |
|  | 11.4 | 10^-1^ | 18.26 | 18.32 | 18.03 | 18.2 | 0.15 |  |  |
|  | 1.14 | 10^-2^ | 21.78 | 21.69 | 21.76 | 21.74 | 0.047 |  |  |
|  | 0.114 | 10^-3^ | 25.1 | 25.14 | 25.55 | 25.26 | 0.25 |  |  |
|  | 0.0114 | 10^-4^ | 29.2 | 29.23 | 29.2 | 29.21 | 0.017 |  |  |
|  | 0.00114 | 10^-5^ | 32.82 | 32.96 | 33.56 | 33.11 | 0.39 |  |  |

**B)**

| **Sample** | **DNA Template Quantity (ng)** | **Dilution** | **Ct values** | | | **Avg** | **SD** | **Efficiency** | | **R^2^** |
| --- | --- | --- | --- | --- | --- | --- | --- | --- | --- | --- |
|  |  |  | **Rep1** | **Rep2** | **Rep3** |  |  |  |  |  |
| PS-HL-F1 Whole Pea | 25 | 10^0^ | 16.97 | 16.99 | 16.98 | 16.98 | 0.01 | 99.2% | 87.8% | 0.9992 |
|  | 2.5 | 10^-1^ | 20.32 | 20.32 | 20.35 | 20.33 | 0.017 |  |  |  |
|  | 0.25 | 10^-2^ | 23.79 | 23.75 | 23.79 | 23.78 | 0.023 |  |  |  |
|  | 0.025 | 10^-3^ | 27.74 | 27.83 | 27.88 | 27.82 | 0.071 |  |  |  |
|  | 0.0025 | 10^-4^ | 31.48 | 31.26 | 31.47 | 31.4 | 0.12 |  |  |  |
|  | 0.00025 | 10^-5^ | 34.74 | 34.79 | 34.57 | 34.7 | 0.12 |  |  |  |

Avg = Average

SD = Standard Deviation

**Supplementary Table S6**: DNA dilutions and corresponding C_t_s for efficiency and linearity calculations. Extractions were complete using the CTAB protocol. A) Processed soy DNA, high starting quantity. B) Processed soy DNA, low starting quantity.

**A)**

| **Sample** | **DNA Template Quantity (ng)** | **Dilution** | **Ct values** | | | **Avg** | **SD** | **Efficiency** | **R^2^** |
| --- | --- | --- | --- | --- | --- | --- | --- | --- | --- |
|  |  |  | **Rep1** | **Rep2** | **Rep3** |  |  |  |  |
| S4.1 Soy Protein Powder Isolate | 100 | 10^0^ | 11.22 | 11.18 | 11.14 | 11.18 | 0.04 | 96.3% | 0.9992 |
|  | 10 | 10^-1^ | 14.59 | 14.57 | 14.62 | 14.59 | 0.025 |  |  |
|  | 1 | 10^-2^ | 17.94 | 17.95 | 17.94 | 17.94 | 0.0058 |  |  |
|  | 0.1 | 10^-3^ | 21.63 | 21.56 | 21.53 | 21.57 | 0.051 |  |  |
|  | 0.01 | 10^-4^ | 26.15 | 26.15 | 26.09 | 26.13 | 0.035 |  |  |
|  | 0.001 | 10^-5^ | 29.79 | 29.89 | 29.9 | 29.86 | 0.061 |  |  |

**B)**

| **Sample** | **DNA Template Quantity (ng)** | **Dilution** | **Ct values** | | | **Avg** | **SD** | **Efficiency** | **R^2^** |
| --- | --- | --- | --- | --- | --- | --- | --- | --- | --- |
|  |  |  | **Rep1** | **Rep2** | **Rep3** |  |  |  |  |
| S4.1 Soy Protein Powder Isolate | 25 | 10^0^ | 13.23 | 13.25 | 13.23 | 13.24 | 0.012 | 97.3% | 0.9997 |
|  | 2.5 | 10^-1^ | 16.71 | 16.64 | 16.69 | 16.68 | 0.036 |  |  |
|  | 0.25 | 10^-2^ | 20.01 | 20.02 | 20 | 20.01 | 0.01 |  |  |
|  | 0.025 | 10^-3^ | 23.62 | 23.66 | 23.62 | 23.63 | 0.023 |  |  |
|  | 0.0025 | 10^-4^ | 27.9 | 27.93 | 27.94 | 27.92 | 0.021 |  |  |
|  | 0.00025 | 10^-5^ | 32.26 | 32.83 | 32.68 | 32.59 | 0.29 |  |  |

Avg = Average

SD = Standard Deviation

**Supplementary Table S7**: DNA dilutions and corresponding C_t_s for efficiency and linearity calculations. Extractions were complete using the CTAB protocol. A) Processed pea DNA, high starting quantity. B) Processed pea DNA, low starting quantity.

**A)**

| **Sample** | **DNA Template Quantity (ng)** | **Dilution** | **Ct values** | | | **Avg** | **SD** | **Efficiency** | | **R^2^** |
| --- | --- | --- | --- | --- | --- | --- | --- | --- | --- | --- |
|  |  |  | **Rep1** | **Rep2** | **Rep3** |  |  |  |  |  |
| P1.1 Soy Protein Powder Isolate | 35 | 10^0^ | 16.2 | 16.18 | 16.2 | 16.19 | 0.012 | 99.2% | 83.4% | 0.998 |
|  | 3.5 | 10^-1^ | 19.53 | 19.54 | 19.58 | 19.55 | 0.026 |  |  |  |
|  | 0.35 | 10^-2^ | 22.98 | 22.98 | 22.98 | 22.98 | 0 |  |  |  |
|  | 0.035 | 10^-3^ | 27.23 | 27.01 | 27.32 | 27.19 | 0.16 |  |  |  |
|  | 0.0035 | 10^-4^ | 31.28 | 31.3 | 31.13 | 31.24 | 0.093 |  |  |  |
|  | 0.00035 | 10^-5^ | 34.26 | 34.44 | 34.03 | 34.24 | 0.21 |  |  |  |

**B)**

| **Sample** | **DNA Template Quantity (ng)** | **Dilution** | **Ct values** | | | **Avg** | **SD** | **Efficiency** | **R^2^** |
| --- | --- | --- | --- | --- | --- | --- | --- | --- | --- |
|  |  |  | **Rep1** | **Rep2** | **Rep3** |  |  |  |  |
| P1.1 Soy Protein Powder Isolate | 15 | 10^0^ | 16.51 | 16.55 | 16.51 | 16.52 | 0.023 | 99.9% | 0.9995 |
|  | 1.5 | 10^-1^ | 19.84 | 19.84 | 19.84 | 19.84 | 0 |  |  |
|  | 0.15 | 10^-2^ | 23.07 | 23.08 | 23.1 | 23.08 | 0.015 |  |  |
|  | 0.015 | 10^-3^ | 27.17 | 27.26 | 27.22 | 27.22 | 0.045 |  |  |
|  | 0.0015 | 10^-4^ | 30.92 | 30.95 | 30.96 | 30.94 | 0.021 |  |  |
|  | 0.00015 | 10^-5^ | 35.56 | 36.5 | 35.11 | 35.72 | 0.71 |  |  |

Avg = Average

SD = Standard Deviation

**Supplementary Table S8**: Raw data (Ct values) of soy target (fresh-derived DNA) and calibrator, with assay-estimated proportion of DNA using the Pfaffl method. Reactions included total template quantity of 5 ng.

| **Expected Sample Percentage** | ***G. max* Probe** | | | **Calibrator Probe** | | | **Proportion** | **Empirical Assay Estimation (%)** |
| --- | --- | --- | --- | --- | --- | --- | --- | --- |
|  | **C_t_ Values** | **Avg** | **SD** | **C_t_ Values** | **Average** | **SD** |  |  |
| 100% | 17.52 | 17.38 | 0.121 | 13 | 12.97 | 0.066 | 1 | 100 |
|  | 17.52 |  |  | 12.89 |  |  |  |  |
|  | 17.31 |  |  | 12.94 |  |  |  |  |
| 99% | 17.18 | 17.22 | 0.0351 | 13 | 12.97 | 0.112 | 1.132 | 113.2 |
|  | 17.25 |  |  | 13.07 |  |  |  |  |
|  | 17.22 |  |  | 12.85 |  |  |  |  |
| 95% | 17.32 | 17.2 | 0.116 | 12.98 | 12.99 | 0.0503 | 1.159 | 115.9 |
|  | 17.18 |  |  | 12.94 |  |  |  |  |
|  | 17.09 |  |  | 13.04 |  |  |  |  |
| 90% | 17.35 | 17.38 | 0.0643 | 13.17 | 12.95 | 0.255 | 0.9923 | 99.23 |
|  | 17.33 |  |  | 12.67 |  |  |  |  |
|  | 17.45 |  |  | 13.01 |  |  |  |  |
| 75% | 17.55 | 17.58 | 0.0252 | 12.95 | 12.96 | 0.0306 | 0.8623 | 86.23 |
|  | 17.58 |  |  | 12.93 |  |  |  |  |
|  | 17.6 |  |  | 12.99 |  |  |  |  |
| 50% | 18.55 | 18.42 | 0.118 | 13.11 | 13.00 | 0.116 | 0.4829 | 48.29 |
|  | 18.39 |  |  | 13.02 |  |  |  |  |
|  | 18.32 |  |  | 12.88 |  |  |  |  |
| 25% | 19.66 | 19.62 | 0.0586 | 13.06 | 12.98 | 0.0802 | 0.2000 | 20 |
|  | 19.64 |  |  | 12.9 |  |  |  |  |
|  | 19.55 |  |  | 12.99 |  |  |  |  |
| 10% | 21.29 | 21.24 | 0.0757 | 13.05 | 13.06 | 0.0557 | 0.06691 | 6.691 |
|  | 21.27 |  |  | 13.01 |  |  |  |  |
|  | 21.15 |  |  | 13.12 |  |  |  |  |
| 5% | 22.63 | 22.56 | 0.0889 | 13.06 | 13.16 | 0.1 | 0.02372 | 2.372 |
|  | 22.59 |  |  | 13.16 |  |  |  |  |
|  | 22.46 |  |  | 13.26 |  |  |  |  |
| 1% | 25.85 | 25.87 | 0.0252 | 12.95 | 13.05 | 0.085 | 0.002236 | 0.2236 |
|  | 25.87 |  |  | 13.11 |  |  |  |  |
|  | 25.9 |  |  | 13.08 |  |  |  |  |

SD = Standard Deviation

**Supplementary Table S9**: Raw data (Ct values) of pea target (fresh-derived DNA) and calibrator, with assay-estimated proportion of DNA using the Pfaffl method. Reactions included total template quantity of 5 ng.

| **Sample Percentage** | ***P. sativum* Probe** | | | **Calibrator Probe** | | | **Proportion** | **Empirical Assay Estimation (%)** |
| --- | --- | --- | --- | --- | --- | --- | --- | --- |
|  | **C_t_ Values** | **Avg** | **SD** | **C_t_ Values** | **Avg** | **SD** |  |  |
| 100% | 18.59 | 18.57 | 0.0245 | 13.03 | 13.13 | 0.106 | 1 | 100 |
|  | 18.56 |  |  | 13.07 |  |  |  |  |
|  | 18.59 |  |  | 13.15 |  |  |  |  |
| 99% | 18.53 | 18.54 | 0.0208 | 12.95 | 13.05 | 0.085 | 0.9656 | 96.56 |
|  | 18.56 |  |  | 13.11 |  |  |  |  |
|  | 18.52 |  |  | 13.08 |  |  |  |  |
| 95% | 18.41 | 18.41 | 0.02 | 13.06 | 13.16 | 0.1 | 1.134 | 113.4 |
|  | 18.43 |  |  | 13.16 |  |  |  |  |
|  | 18.39 |  |  | 13.26 |  |  |  |  |
| 90% | 18.35 | 18.37 | 0.0404 | 13.05 | 13.06 | 0.0557 | 1.115 | 111.5 |
|  | 18.35 |  |  | 13.01 |  |  |  |  |
|  | 18.42 |  |  | 13.12 |  |  |  |  |
| 75% | 18.65 | 18.65 | 0 | 13.06 | 12.98 | 0.0802 | 0.8584 | 85.84 |
|  | 18.65 |  |  | 12.9 |  |  |  |  |
|  | 18.65 |  |  | 12.99 |  |  |  |  |
| 50% | 19.12 | 19.13 | 0.0231 | 13.11 | 13.00 | 0.116 | 0.6333 | 63.33 |
|  | 19.16 |  |  | 13.02 |  |  |  |  |
|  | 19.12 |  |  | 12.88 |  |  |  |  |
| 25% | 20.73 | 20.74 | 0.0208 | 12.95 | 12.96 | 0.0306 | 0.2138 | 21.38 |
|  | 20.72 |  |  | 12.93 |  |  |  |  |
|  | 20.76 |  |  | 12.99 |  |  |  |  |
| 10% | 25.22 | 25.45 | 1.916 | 13.17 | 12.95 | 0.255 | 0.009552 | 0.9552 |
|  | 27.48 |  |  | 12.67 |  |  |  |  |
|  | 23.67 |  |  | 13.01 |  |  |  |  |
| 5% | 29.97 | 30.02 | 0.279 | 12.98 | 12.99 | 0.0503 | 0.0004852 | 0.04852 |
|  | 30.33 |  |  | 12.94 |  |  |  |  |
|  | 29.78 |  |  | 13.04 |  |  |  |  |
| 1% | 40 | 40* | 0* | 13 | 12.97 | 0.112 | 6.824E-7 | 6.824E-5 |
|  | 40 |  |  | 13.07 |  |  |  |  |
|  | 40 |  |  | 12.85 |  |  |  |  |

SD = Standard Deviation

*Ct values of 40 are not precise measurements. The LightCycler software assigns a C_t_ value of 40 if C_t_ ≥ 40.

**Supplementary Table S10**: Raw data (Ct values) of soy target (powder-derived DNA) and calibrator, with assay-estimated proportion of DNA using the Pfaffl method. Reactions included total template quantity of 5 ng.

| **Sample Percentage** | ***G. max* Probe** | | | **Calibrator Probe** | | | **Proportion** | **Empirical Assay Estimation (%)** |
| --- | --- | --- | --- | --- | --- | --- | --- | --- |
|  | **C_t_ Values** | **Average** | **SD** | **C_t_ Values** | **Average** | **SD** |  |  |
| 100% | 15.62 | 15.61 | 0.0337 | 11.89 | 11.93 | 0.0545 | 1 | 100 |
|  | 15.63 |  |  | 12 |  |  |  |  |
|  | 15.63 |  |  | 11.88 |  |  |  |  |
| 99% | 15.63 | 15.65 | 0.0289 | 12 | 12.01 | 0.0321 | 1.036 | 103.6 |
|  | 15.68 |  |  | 11.99 |  |  |  |  |
|  | 15.63 |  |  | 12.05 |  |  |  |  |
| 95% | 15.68 | 15.7 | 0.0208 | 12.04 | 11.96 | 0.0681 | 0.9682 | 96.82 |
|  | 15.69 |  |  | 11.94 |  |  |  |  |
|  | 15.72 |  |  | 11.91 |  |  |  |  |
| 90% | 15.74 | 15.74 | 0.00577 | 11.92 | 11.95 | 0.0462 | 0.9276 | 92.76 |
|  | 15.75 |  |  | 11.92 |  |  |  |  |
|  | 15.74 |  |  | 12 |  |  |  |  |
| 75% | 15.94 | 15.98 | 0.0379 | 11.99 | 12.01 | 0.0379 | 0.8220 | 82.2 |
|  | 16.01 |  |  | 11.98 |  |  |  |  |
|  | 16 |  |  | 12.05 |  |  |  |  |
| 50% | 16.63 | 16.65 | 0.02 | 11.99 | 11.99 | 0.0252 | 0.5195 | 51.95 |
|  | 16.67 |  |  | 11.97 |  |  |  |  |
|  | 16.65 |  |  | 12.02 |  |  |  |  |
| 25% | 17.77 | 17.78 | 0.0755 | 12.01 | 12.02 | 0.0321 | 0.2475 | 24.75 |
|  | 17.71 |  |  | 12 |  |  |  |  |
|  | 17.86 |  |  | 12.06 |  |  |  |  |
| 10% | 19.37 | 19.36 | 0.0755 | 12.15 | 12.09 | 0.06 | 0.08467 | 8.467 |
|  | 19.43 |  |  | 12.03 |  |  |  |  |
|  | 19.28 |  |  | 12.09 |  |  |  |  |
| 5% | 20.64 | 20.72 | 0.07 | 12.11 | 12.1 | 0.0115 | 0.03270 | 3.27 |
|  | 20.75 |  |  | 12.09 |  |  |  |  |
|  | 20.77 |  |  | 12.09 |  |  |  |  |
| 1% | 25.05 | 25.13 | 0.108 | 12.26 | 12.25 | 0.0264 | 0.002036 | 0.2036 |
|  | 25.08 |  |  | 12.27 |  |  |  |  |
|  | 25.25 |  |  | 12.22 |  |  |  |  |

SD = Standard Deviation

**Supplementary Table S11**: Raw data (Ct values) of pea target (powder-derived DNA) and calibrator, with assay-estimated proportion of DNA using the Pfaffl method. Reactions included total template quantity of 5 ng.

| **Sample Percentage** | ***P. sativum* Probe** | | | **Calibrator Probe** | | | **Proportion** | **Empirical Assay Estimation (%)** |
| --- | --- | --- | --- | --- | --- | --- | --- | --- |
|  | **C_t_ Values** | **Average** | **SD** | **C_t_ Values** | **Average** | **SD** |  |  |
| 100% | 17.39 | 17.34 | 0.0608 | 12.16 | 12.08 | 0.0560 | 1 | 100 |
|  | 17.4 |  |  | 12.03 |  |  |  |  |
|  | 17.29 |  |  | 12.06 |  |  |  |  |
| 99% | 17.31 | 17.3 | 0.0321 | 12.26 | 12.25 | 0.0264 | 1.159 | 115.9 |
|  | 17.26 |  |  | 12.27 |  |  |  |  |
|  | 17.32 |  |  | 12.22 |  |  |  |  |
| 95% | 17.22 | 17.23 | 0.0173 | 12.11 | 12.1 | 0.0115 | 1.093 | 109.3 |
|  | 17.25 |  |  | 12.09 |  |  |  |  |
|  | 17.22 |  |  | 12.09 |  |  |  |  |
| 90% | 17.21 | 17.2 | 0.0153 | 12.15 | 12.09 | 0.06 | 1.057 | 105.7 |
|  | 17.2 |  |  | 12.03 |  |  |  |  |
|  | 17.18 |  |  | 12.09 |  |  |  |  |
| 75% | 17.35 | 17.36 | 0.0173 | 12.01 | 12.02 | 0.0321 | 0.9505 | 95.05 |
|  | 17.35 |  |  | 12 |  |  |  |  |
|  | 17.38 |  |  | 12.06 |  |  |  |  |
| 50% | 17.92 | 17.94 | 0.0173 | 11.99 | 11.99 | 0.0252 | 0.6244 | 62.44 |
|  | 17.95 |  |  | 11.97 |  |  |  |  |
|  | 17.95 |  |  | 12.02 |  |  |  |  |
| 25% | 19.35 | 19.5 | 0.145 | 11.99 | 12.01 | 0.0379 | 0.2155 | 21.55 |
|  | 19.64 |  |  | 11.98 |  |  |  |  |
|  | 19.5 |  |  | 12.05 |  |  |  |  |
| 10% | 25.48 | 25.62 | 0.289 | 11.92 | 11.95 | 0.0462 | 0.003028 | 0.3028 |
|  | 25.44 |  |  | 11.92 |  |  |  |  |
|  | 25.96 |  |  | 12 |  |  |  |  |
| 5% | 28.82 | 29.02 | 0.244 | 12.04 | 11.96 | 0.0681 | 0.0002961 | 0.02961 |
|  | 28.94 |  |  | 11.94 |  |  |  |  |
|  | 29.29 |  |  | 11.91 |  |  |  |  |
| 1% | 40 | 40* | 0* | 12 | 12.01 | 0.0321 | 1.582E-07 | 1.582E-05 |
|  | 40 |  |  | 11.99 |  |  |  |  |
|  | 40 |  |  | 12.05 |  |  |  |  |

SD = Standard Deviation

*Ct values of 40 are not precise measurements. The LightCycler software assigns a C_t_ value of 40 if C_t_ ≥ 40.
